# Understanding uncertainties in contemporary and future extreme wave events for broad-scale impact and adaptation planning

**DOI:** 10.1126/sciadv.ade3170

**Published:** 2023-01-11

**Authors:** Joao Morim, Thomas Wahl, Sean Vitousek, Sara Santamaria-Aguilar, Ian Young, Mark Hemer

**Affiliations:** ^1^ Univeristy of Central Florida (UCF), Orlando, FL, USA.; ^2^ Pacific Coastal and Marine Science Center, U.S. Geological Survey (USGS), Santa Cruz, CA, USA.; ^3^ Department of Infrastructure Engineering, University of Melbourne, Parkville, Victoria, Australia.; ^4^ Commonwealth Scientific and Industrial Research Organisation (CSIRO) Oceans and Atmosphere, Hobart, Tasmania, Australia.

## Abstract

Understanding uncertainties in extreme wind-wave events is essential for offshore/coastal risk and adaptation estimates. Despite this, uncertainties in contemporary extreme wave events have not been assessed, and projections are still limited. Here, we quantify, at global scale, the uncertainties in contemporary extreme wave estimates across an ensemble of widely used global wave reanalyses/hindcasts supported by observations. We find that contemporary uncertainties in 50-year return period wave heights ($H_{s}^{50}$) reach (on average) ~2.5 m in regions adjacent to coastlines and are primarily driven by atmospheric forcing. Furthermore, we show that uncertainties in contemporary $H_{s}^{50}$ estimates dominate projected 21st-century changes in $H_{s}^{50}$ across ~80% of global ocean and coastlines. When translated into broad-scale coastal risk analysis, these uncertainties are comparable to those from storm surges and projected sea level rise. Thus, uncertainties in contemporary extreme wave events need to be combined with those of projections to fully assess potential impacts.

## Teaser

Uncertainties in extreme wave events are critical for coastal risk and adaptation analysis for present and future climates.

## INTRODUCTION

Extreme waves are a key driver of coastal change (*1*) and loss of natural coastal wetlands (*2*) and a major contributor to coastal flooding over multiple time scales (*3*, *4*), with wave run-up often representing up to ~50% of extreme total water levels along many coastlines (*3*, *5*). These events can also disrupt shipping (*6*) and are critical to establish design limits for offshore and coastal infrastructure (e.g., natural gas and oil drilling platforms, aquaculture farms, renewable energy projects, and coastal defenses) (*7*, *8*), which are forecasted to expand by up to ~50% within less than a decade (*9*, *10*). Thus, estimating and understanding uncertainties in extreme wave events for the present-day climate is critical to support global offshore and coastal developments (*10*), assess hazards and adaptation measures (*11*), and substantiate projections under future climate scenarios (*12*).

At broad spatial scales, multidecadal wave reanalysis and hindcast model products are needed for impact assessments as wave buoy records are sparsely distributed globally and have limited length, and satellite altimetry observations suffer from relatively low temporal resolution (*13*). The contribution of extreme waves to structural design loads, erosion, and flooding (via wave setup and/or run-up) is usually determined as a function of deep-water significant wave height (*H_s_*) (*14*–*20*). Now, multiple contemporary global wave reanalysis and hindcast products, generated using different calibration data, numerical spectral wave models, and/or atmospheric (reanalysis) forcing, are being used to generate such estimates (*16*–*21*). Comparative analyses show that offshore/coastal hazard modeling estimates can change significantly depending on datasets and models adopted (*22*), which can affect policy and adaptation measures (*22*). Some former analyses, comparing specific historical years and/or specific hindcast data, suggest that extreme wave heights could also vary considerably depending on the global wave product (*23*–*25*). Nevertheless, a comprehensive global-scale uncertainty analysis of contemporary extreme *H_s_* estimates across multiple widely used wave reanalysis/hindcast products is still missing, as previously acknowledged (*22*, *26*).

In addition, increasing evidence suggests that extreme waves are likely to considerably change across many global ocean areas and coastlines due to climate change (*27*–*29*), and such changes need to be accounted for when determining offshore and coastal impacts (*30*). For example, in the Southern Hemisphere, low-probability extreme wave events obtained using annual maxima (AMAX) *H_s_* have been projected to increase by up to ~15% by the end of the century (*31*–*34*). However, existing projections of extreme *H_s_* rely on single-method wave ensembles (*27*, *28*, *31*, *33*, *34*) and thus neglect any uncertainties between different statistical and/or dynamical wave simulations (*26*), which are known to account for up to ~50% of the total projection uncertainty (*26*). These projections have also been substantiated on the basis of different global wave reanalysis and hindcast model datasets (*35*). Thus, there is a need for an all-encompassing analysis of projected changes in extreme *H_s_* events.

Understanding uncertainties in extreme wave events for the present-day climate and comparing them to potential future changes and associated uncertainties due to global climate warming are thereby critical to support planning and adaptation strategies (*22*, *36*). Here, we quantify present-day uncertainties in extreme *H_s_* estimates using a novel ensemble of state-of-the-art global wave model products (*37*) and compare such estimates against those obtained from 64 wave buoys around the world. Furthermore, we compare the present-day uncertainties in extreme *H_s_* estimates to projected future changes in extreme *H_s_* and associated uncertainties obtained from the most comprehensive ensemble of global wave projections developed to date.

## RESULTS

### Extreme value analysis

To characterize extreme wave events, we use the AMAX approach and apply the generalized extreme value (GEV) and Gumbel (GUM) extreme value distributions, which are widely used to estimate the *n*-year return period *H_s_* (henceforward $H_{s}^{n}$) required for offshore and coastal engineering designs (*31*–*34*). Other extreme value analysis (EVA) methods exist (e.g., peaks over threshold, *r*-largest, and conditional average exceedance rate) (*38*–*40*) but require hourly or sub-daily time series *H_s_* data, which are not archived (and/or accessible) across the full global wave product ensembles (Materials and Methods; tables S1 to S3) due to computational and storage constraints. We estimate present-day $H_{s}^{n}$ values and their confidence intervals by fitting these two extreme value distributions to the time series of AMAX *H_s_* (GEV-AMAX and GUM-AMAX for GEV and GUM, respectively) from 12 global wave model products that span the analysis period of 35 years (1980 to 2014) (Materials and Methods). Two global wave products are wind-wave reanalyses derived using fully coupled atmosphere-wave models that assimilate satellite wave data (from 1991 onward) to adjust model predicted wave spectra. The other products are global wave hindcasts generated, at different research institutes, by directly forcing global spectral wave models with surface winds from different atmospheric reanalyses (table S1). Through this analysis, we use GUM-MAX (as reference) because it provides an overall, more suitable model for the AMAX *H_s_* databases from both observations and model products over most of the global ocean (Materials and Methods), consistent with previous work (*33*). However, we still consider GEV-AMAX to assess uncertainties associated with the extreme value model used, as later discussed.

### Comparison with observations

**Fig. 1.**
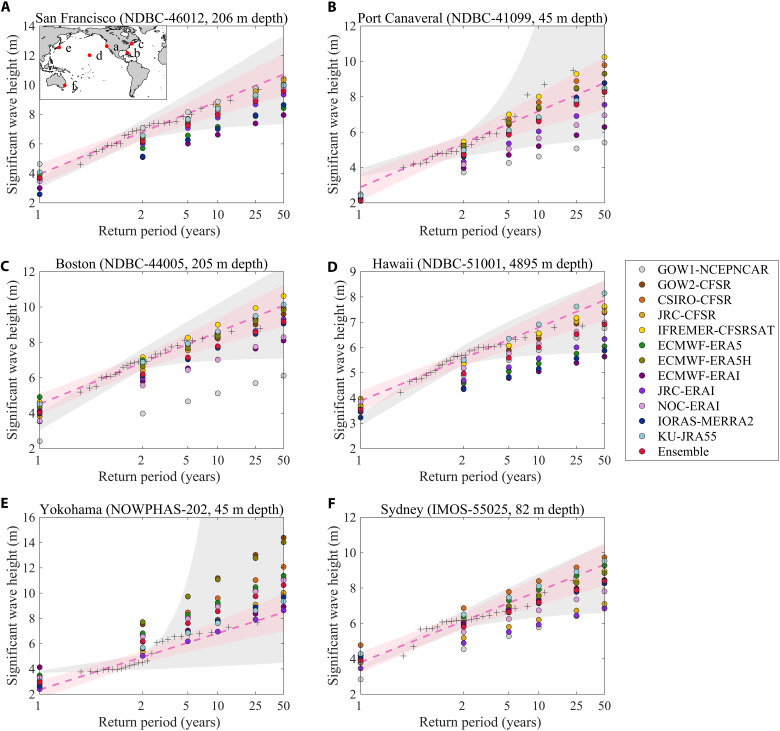
Return period significant wave height ($H_{s}^{n}$) estimates for representative wave buoy sites off major coastal cities. (**A** to **F**) The plotting positions (“+”) were obtained from the observed AMAX for each site and are hence directly comparable to the GUM-AMAX fit from the different global wave products (see circles). Shaded pink (gray) bands represent the 95% confidence limits of GUM-AMAX (GEV-AMAX) method applied to the wave observations, and the dashed line is the GUM-AMAX’s central estimate. The ID code for each wave buoy station is provided within each subpanel (table S2).

Model estimates of $H_{s}^{n}$ (from GUM-AMAX) are compared with the estimates derived from 64 moored wave-buoy stations with suitable locations, record lengths, and completeness (Materials and Methods; fig. S1). In this analysis, we limit estimates to 50-year return period events (or $H_{s}^{50}$) to avoid large uncertainties associated with extrapolation to return periods far beyond the length of the wave observational records and global wave datasets used (tables S1 and S2). The comparison of return levels from observations and products shows that the spread in present-day $H_{s}^{n}$ estimates (from different global wave model products) can be considerable and is highly variable depending on ocean regions and locations, as shown for six representative (extra-tropical and tropical) sites near major coastal cities (Fig. 1). Furthermore, spread values in present-day $H_{s}^{n}$ product estimates become particularly larger for those events with relatively lower probability and higher-risk potential, such as $H_{s}^{20}$ and longer. For instance, estimates for $H_{s}^{50}$ differ by nearly 5 m off Boston, Port Canaveral, and Yokohama and 3 m off Hawaii, San Francisco, and Sydney. We also find similar spread at other representative sites (fig. S2).

**Fig. 2.**
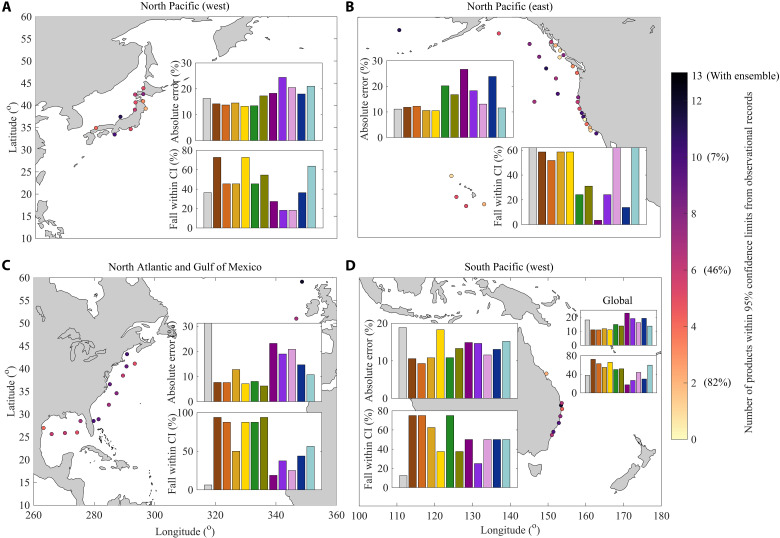
Number of global wave product estimates falling within the 95% confidence limits of the observations for $H_{s}^{50}$ at all wave buoy sites. (**A**) North Pacific (east), (**B**) North Pacific (west), (**C**) North Atlantic and Gulf of Mexico, and (**D**) South Pacific (west). The column chart colors (within each subplot) are consistent with the legend of Fig. 1. The percentage of sites with more than 2, 6, and 10 model estimates falling within the observational confidence intervals (CI) is also provided within brackets along the color bar scale. For each respective basin, the mean absolute error across all locations and all products is shown along with the percentage of sites that each product falls within the observational bounds.

When the central estimates from the global wave products are compared to the 95% confidence intervals of the GUM-AMAX estimates from the observations, we find a significant percentage of sites where 6 or more models lie within observational limits for the 50-year events (~45%) and a small percentage of sites where 10 or more models fall within the observational intervals (~7%) (Fig. 2). Furthermore, we find that no individual global wave model product leads to the lowest or highest errors compared to the observations at all sites or even within specific regions (figs. S3 and S4). Such results, along with the sparsity of global wave buoy records, preclude any weighting of individual ensemble model products based on their relative skill (Materials and Methods). Although there is a general tendency of global wave products to underestimate $H_{s}^{50}$ (figs. S5 and S6), consistent with spectral wave models underestimating storm peak *H_s_* (*23*, *24*, *41*), absolute errors relative to wave observations change significantly depending on product and location. However, some products result in smaller mean absolute errors across all locations and fall within the 95% confidence intervals of the observational records across many more locations (Fig. 2). For instance, all Climate Forecast System Reanalysis (CFSR)–based global wave products show much smaller mean absolute errors (~10%) and also lie within the observational limits far more often (~60%) compared to ECMWF-ERAI (23 and 18%), IORAS-MERRA2 (19 and 30%), and GOW1-NCEPNCAR (18 and 37%), respectively. The overlap between confidence bounds of GUM-AMAX estimates from model products and observational records was also assessed. However, at many locations, the confidence bounds estimated from both datasets are relatively large and therefore do not provide a faithful measure of how well model products represent observations.

### Clustering analysis

**Fig. 3.**
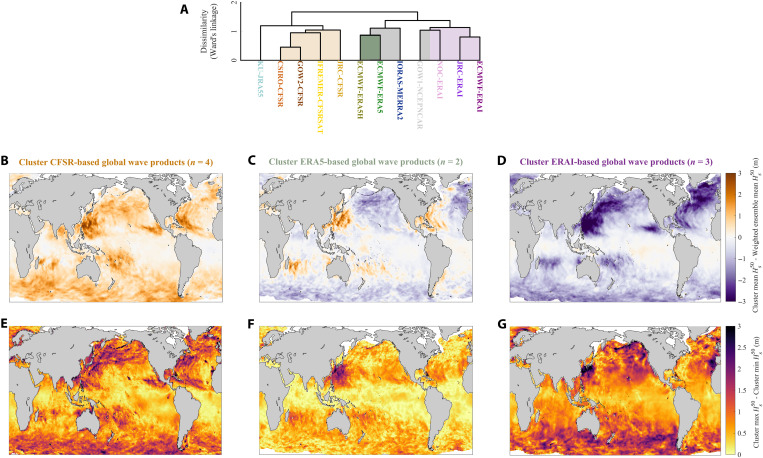
Cluster analysis of $H_{s}^{50}$ estimates obtained from global wave model products. (**A**) Cluster diagram (dendrogram) resulting from Euclidean distance–based Ward’s minimum variance clustering using global pairwise $H_{s}^{50}$ (Materials and Methods) with the vertical axis representing the distance or dissimilarity between clusters and cluster members presented as log scale for clarity. Shading represents defined clusters and subclusters. Note that gray shading on the cluster diagram represents subcluster(s) with different atmospheric forcing within one of the main clusters (**B** to **D**) Mean estimate of each cluster, or subcluster, based on the same atmospheric reanalysis, as per colors, minus the weighted ensemble mean. (**E** to **G**) Maximum difference between estimates within each respective cluster or subcluster.

We show that extreme $H_{s}^{50}$ estimates from different global wave products (fig. S6) are clustered by atmospheric reanalysis forcing (Fig. 3). Our cluster analysis (see Materials and Methods) defines three key groups (Fig. 3A) with products using the same reanalysis forcing falling within the same cluster regardless of global wind-wave modeling method used (e.g., source-term wave parameterization, numerical resolution, and/or spectral frequency). Hence, global wave products generated using the same atmospheric reanalysis forcing lead to estimates of similar magnitude and spatial pattern (fig. S7), highlighting the strong influence of surface winds on extreme ocean wave estimates (*41*, *42*). For example, all CFSR-based wave model products lead to higher estimates, almost everywhere, compared to ERAI-driven products (see Fig. 3B). The sample space available within each cluster (see Fig. 3) precludes from quantifying within-cluster similarities and separates the influence of different global wind-wave modeling methods on extreme wave estimates. However, we still show that, even within each cluster or subcluster based on the same reanalysis forcing (e.g., CFSR or ERAI cluster), differences in contemporary $H_{s}^{50}$ estimates due to wave modeling methodology (e.g., numerical scheme, resolution, spectral frequency, and/or source-term wave parameterizations) can reach up to ~3 m across widespread ocean regions (Fig. 3C).

### Uncertainty analysis

**Fig. 4.**
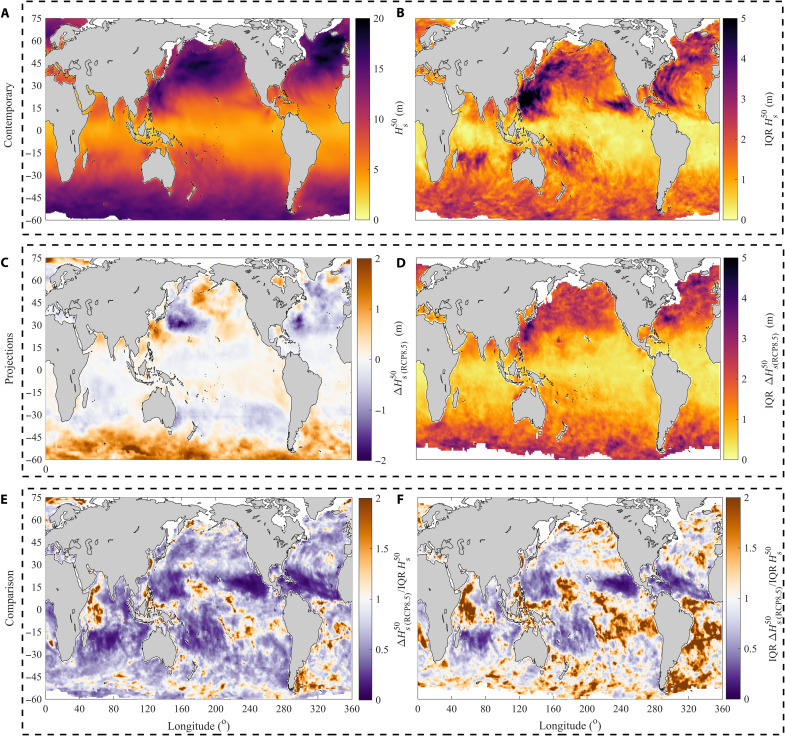
Comparison of present-day uncertainty, projected future changes, and projection uncertainty for $H_{s}^{50}$. (**A**) Weighted ensemble mean of contemporary ensemble (hindcasts and reanalysis products) estimates ($H_{s}^{50}$). (**B**) Interquartile range (IQR) of contemporary ensemble (hindcasts and reanalysis products) estimates (IQR $H_{s}^{50}$). (**C**) Weighted ensemble mean of projected future changes ($\Delta H_{s\;\mathrm{RCP}8.5}^{50}$) using climate model–driven wave simulations. (**D**) IQR of ensemble projections (IQR $\Delta H_{s\;\mathrm{RCP}8.5}^{50}$). (**E**) Weighted ensemble mean (C)/(B) [and rather than weighted ensemble mean (C) over (B)]. (**F**) Weighted ensemble mean (D)/(B) [and rather than weighted ensemble mean (D) over (B)].

We calculate the weighted ensemble mean (by forcing) of contemporary $H_{s}^{50}$ (Materials and Methods) across all the global wave model products (Fig. 4A) and quantify its associated uncertainty using the ensemble interquartile range (IQR) (Fig. 4B). Our results show that IQR values exceed 1 m (and 2 m) for 50 and 79% (32 and 13%) of global ocean and coastlines, respectively (Table 1). Spatially, IQR values can extend from less than ~1 m within tropical areas up to ~3 m across widespread extratropical cyclone (ETC) areas and 5 m across tropical cyclone (TC)–dominated areas (Fig. 4B). The maximum difference between ensemble estimates ranges from ~3 up to ~7 m across most nontropical areas (fig. S8). Attributing the potential underlying causes for these uncertainties across the globe is beyond the analysis. However, we believe that they are largely associated with the representation of ETC and TC systems within the reanalysis datasets, given that atmospheric forcing is the major source of uncertainty among contemporary extreme *H_s_* estimates (Fig. 3). Although ETC and TC intensities are generally underrepresented within most atmospheric reanalysis datasets relative to observational datasets, particularly TC events (*43*, *44*), specific atmospheric reanalyses can capture and resolve certain features of ETC and TC systems, such as cyclone storm tracks and/or frequency (e.g., owing to improved model resolution, data assimilation, and/or bias-correction methods) (*43*–*47*). Hence, some reanalysis-forced global wave products are more capable of resolving specific features of ETC- and/or TC-generated wave events than others (fig. S9), leading to a considerable spread among extreme wave estimates across such regions (Fig. 4B).

**Table 1.** Uncertainty comparison across existing marine-built infrastructure sites, global ocean area and coastline length. See Materials and Methods for calculation of uncertainties.

Percentage of	Constructed offshore and coastal infrastructure				
	Gas and oil	Wind farms	Ports	Global coastline length	Global ocean area
	(267)	(26)	(1045)		
Present-day uncertainty (IQR) > 1 m (2 m)	41.2% (7.9%)	78.13% (0%)	49.6% (11.3%)	50.1% (12.5%)	79.7% (31.5%)
Projection uncertainty (IQR) > 1 m (2 m)	49.8% (4.9%)	36.0% (4.0%)	41.3% (6.4%)	43.6% (7.1%)	56.1% (24.5%)
Combined uncertainty > 1 m (2 m)	67.0% (22.1%)	100.0% (11.5%)	73.0% (27.9%)	75.0% (26.9%)	74.9% (48.9%)
Present-day uncertainty (IQR) > Projected absolute changes (∆)	69.0%	85.0%	80.0%	79.0%	81.4%
Present-day uncertainty (IQR) > Projection uncertainty (IQR)	48.3%	69.0%	61.0%	58.8%	52.0%

To date, much research has been conducted to assess uncertainties associated with EVA models used to parameterize extreme events. For example, 100-year storm surge estimates can vary by more than 0.5 m (*48*) and 100-year *H_s_* estimates by up to ~0.5 to 1 m depending on location (*38*). Comparison of present-day $H_{s}^{50}$ uncertainty due to global wave product differences with those from the selection of EVA model (here by comparing GEV-AMAX and GUM-AMAX) shows that the former dominates over the latter (almost everywhere) across the globe (fig. S10). We note, however, that we only considered two commonly used EVA methods within our analysis as previously discussed, and extending this analysis to sample more methods, such as *r*-largest, peak over threshold, and/or conditional average exceedance rate, could result in an increase in the EVA-related uncertainty (and exacerbate the combined uncertainties presented in this analysis).

### Extreme value projections

So far, we analyzed present-day uncertainty associated with contemporary extreme *H_s_* estimates from global wave reanalysis/hindcast products forced with atmospheric reanalysis winds. Next, we compare this uncertainty to projected future changes in extreme wave events and associated uncertainties due to global warming, obtained using statistical and dynamical wave simulations forced with climate model wind projections. To do this, and for consistency, we assess changes in $H_{s}^{n}$ using AMAX *H_s_* data extracted from the most comprehensive ensemble of global wave projections containing a total of 39 global wave simulations (*49*) developed for the high-risk representation concentration pathway RCP8.5 (Materials and Methods) (*50*). In contrast to past analyses, which calculated projected changes in extreme wave events based on single-method ensembles (*27*, *31*–*33*, *35*), our ensemble (*26*, *49*) covers different climate model, global climate models (GCM), forcing and wave-modeling methods, hence providing the most complete assessment of uncertainty to date (*49*). The projected future changes are obtained using a weighted multimember ensemble mean and its associated uncertainty (Fig. 4, C and D) quantified by bootstrapping the ensemble IQR (Materials and Methods). We find that $H_{s}^{50}$ values are projected to increase by ~5 to 15% across the Southern Ocean, eastern Pacific Ocean, and northeastern Pacific Ocean and also across localized regions (e.g., Arabian Sea, Gulf of Bengal, Aleutian Sea, or China Sea). In contrast, there is a widespread projected decrease of up to −15% across the northern and central Atlantic Ocean, northwestern Pacific Ocean, Indian Ocean, and southern Pacific Ocean. In general, the overall spatial patterns of change in $H_{s}^{50}$ shown are consistent with those of past analyses based on CMIP5-based dynamical wave ensembles (*27*, *33*).

When we compare the present-day uncertainties to projected changes for the 50-year event, we find that the former dominates over the latter across ~81% of the global ocean and ~79% of the coastline (Table 1 and Fig. 4E). The exceptions are localized tropical areas where the present-day uncertainty values are almost negligible (Fig. 4B) and a few particular areas within the Southern Ocean where projected future changes are large enough to exceed the present-day uncertainties (see fig. S4). In addition, we find that the present-day uncertainties exceed the uncertainties in the projections across ~52% of the global ocean area and ~59% of the coastline (Table 1). These areas are predominantly TC-dominated regions (Fig. 4F) but extend to specific extratropical areas (e.g., central-northern Atlantic Ocean, Southern Ocean, and southeastern Pacific Ocean) and to some localized regions (e.g., Gulf of Guinea). In all remaining regions, the uncertainties in the future projections exceed the present-day uncertainties.

The projected future changes and future uncertainties presented within regions directly affected by TC activity need to be considered carefully. We attribute the low ratio (<0.25) shown within TC-dominated regions (see Fig. 4, E and F) to the limited representation of intense TC systems within standard GCM simulations. So far, only a few GCM-driven global wave simulations have been forced with atmospheric forcing high-resolution models (*51*), instead being forced with GCMs that tend to underestimate TC intensity and/or frequency due to their coarser resolutions (*52*). Therefore, most GCM-driven global wave simulations underestimate extreme $H_{s}^{n}$ patterns produced by TC events (fig. S11) and cannot sufficiently resolve their potential future change due to global warming (*53*, *54*). In addition, although our ensemble of projections exhibits no robust (or statistically significant) changes in the shape of the underlying distribution (fig. S12) (*33*), GUM-AMAX usually underestimates TC-driven wave extremes (Materials and Methods), which are generally characterized by heavy-tailed distributions (fig. S9) and more adequately resolved using a “nonzero” shape distribution (such as GEV-AMAX). Now, there is no consensus on anthropogenic influences on major TC events (*55*) and TC-driven wave extremes—simulated using high-resolution atmospheric forcing (*53*, *54*). However, if proven that global warming could drive a significant increase in major TC events not resolved within existing simulations, then this could potentially affect the shape of the underlying distribution and make projected future changes and projection uncertainties potentially comparable, or even exceed, the present-day uncertainties within those TC areas (further exacerbating the combined uncertainties discussed within this analysis).

**Fig. 5.**
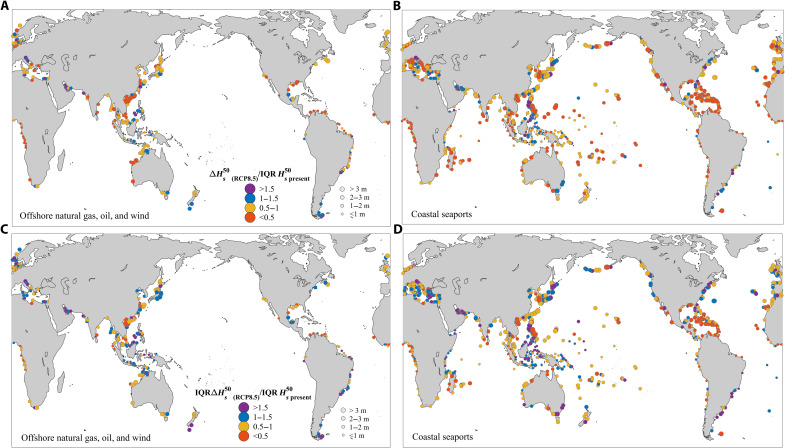
Relative importance of present-day $H_{s}^{50}$ uncertainties and projected $H_{s}^{50}$ changes, and associated uncertainties, at existing offshore and coastal infrastructure locations. (**A**) Relative importance of projected absolute changes relative to present-day climate uncertainties at offshore oil and natural gas platforms (circles) and offshore wind farms (squares). (**B**) Relative importance of projected absolute changes relative to present-day climate uncertainties at open-coast sea ports. (**C**) Relative importance of projection uncertainty relative to present-day climate uncertainties at offshore oil and gas platforms (circles) and offshore wind farms (squares) and (**D**) relative importance of projection uncertainty relative to present-day uncertainties at open-coast sea ports. In all panels, combined uncertainties are indicated by the circle sizes according to the legends of (A) and (C).

To further contextualize our findings, we present results at existing global offshore oil and gas platforms, offshore wind projects, and coastal seaports (see Fig. 5). Figure 5 (A and B) shows that present-day uncertainties for the 50-year events exceed projected future changes (dark and light orange marks), sometimes by more than 50% (dark orange marks), at more than ~70% of the infrastructure locations (Table 1). However, at 30% of the offshore and gas platform sites, projected changes exceed present-day uncertainties, highlighting that many existing infrastructure supporting the offshore energy production industry are at greater risk of being affected by changes in extreme waves due to global climate warming. Figure 5 (C and D) shows that present-day uncertainties exceed the uncertainties in global wave projections (purple and blue marks) at a significant percentage of infrastructure sites (48% of offshore oil and natural gas platforms, 69% of offshore wind farms, and 61% of open-coast seaports). The combined uncertainties are shown to be considerable at many sites (Fig. 4, C and D), exceeding more than 1 m (and 2 m) at 67% (22.1%) of the global offshore platform locations and 73% (27%) of coastal seaport sites (Table 1). Consistent with Fig. 4, we find that results have strong regional dependence. For example, most sites in Africa and South America exhibit a relatively low combined uncertainties (<1 m) (yet still dominated by present-day uncertainties), while sites in Europe and North America have relatively higher uncertainties (>2 to 3 m).

## DISCUSSION

Our analyses have shown large uncertainties associated with present-day extreme $H_{s}^{n}$ estimates determined from many global wave products that are widely used for broad-scale offshore and coastal infrastructure design and hazard/risk assessments. Consequently, findings drawn from any single global wave product need to be treated carefully and/or contextualized. In addition to showing that atmospheric (reanalysis) forcing is the major driver of uncertainties in present-day extreme $H_{s}^{n}$ estimates, our analysis also indicates that differences in global wave modeling methods can lead to significant discrepancies between present-day $H_{s}^{n}$ estimates. This analysis also highlight that no individual product can represent the observational records across all wave buoy locations even within specific regions. Instead, within each region or basin with available buoy observations, some specific products lead to a more reliable representation of the regional buoy records than others.

Our analysis assesses projected changes in $H_{s}^{n}$ using a large CMIP5-coordinated ensemble of wave projections across climate models and global wave modeling methods (*49*), thus allowing a much improved sampling of uncertainty relative to past analyses (*27*, *28*, *31*, *33*, *34*). We show that present-day extreme $H_{s}^{n}$ uncertainties largely outpace climate-driven changes in $H_{s}^{n}$ almost everywhere, even when considering a high-emission scenario (5°C warming by 2100) with no stringent climate mitigation (RCP8.5) (*50*). In addition, present-day $H_{s}^{n}$ uncertainty levels are found to be comparable to and/or exceed the uncertainties associated with projected future $H_{s}^{n}$ changes across widespread areas. These results suggest that present-day uncertainties are even more relevant when considering milder future warming climate scenarios (ranging 2° to 4°C) that consider stricter climate mitigation policies.

The uncertainties in present-day extreme $H_{s}^{n}$ estimates shown have wide-reaching implications for structural designs loads, coastal erosion, and flooding. For example, although different wave setup parameterizations have been used (*18*, *19*, *56*), some large-scale coastal flood risk analyses simply approximate wave setup contribution as ~20% of offshore extreme *H_s_* (*16*, *17*, *57*–*60*). On the basis of our results, which show that present-day $H_{s}^{50}$ uncertainty owing to global wave model product differences can reach ~2.5 m when averaged across data offshore of coastal areas and up to 5 m at specific locations, this could lead to uncertainties of 0.5 m and up to 1 m at specific sites. This exceeds or is comparable to other uncertainties that are considered critical for current and future coastal hazard flooding and adaptation assessments (*22*), such as global tide-storm surge hindcast models, digital elevation model datasets, and sea level rise scenarios (*22*, *48*). For instance, contemporary extreme storm surge estimates (e.g., 100-year events) differ on average by 0.5 m, depending on the global hindcast model used (*48*). Although broad-scale assessments are now beginning to consider potential changes in extreme wave climate due to climate change (*16*–*19*), assessments to date have not accounted for present-day uncertainties in extreme wave events, which are essential to fully assess potential hazards and adaptation needs.

Expansion of the existing buoy networks and remote sensing datasets will improve assessment of extreme ocean wave events from global wave model products and potentially enhance future products (*13*, *61*). The Sofar network of globally distributed drifting surface weather buoys has expanded rapidly to more than 600 buoys (*62*). Maintaining such networks to enable climate relevant over time will provide data with greatly improved spatial resolution to complement the network of fixed observation platforms. Improving representation of TC and ETC systems within global atmospheric reanalyses with more data assimilation (*63*, *64*) and development of new globally downscaled atmospheric reanalysis data (*65*) would also help to further constrain uncertainties. Expanding the limited number of dynamical global wave simulations forced by high-resolution downscaled climate models (0.25° or less) that are more capable of resolving TC-driven waves (relative to coarse CMIP-driven global wave simulations) (*53*, *54*) might also help to understand and reduce uncertainties in ETC and TC areas. As previously discussed, our community-based ensemble of global wave projections [and any other ensembles based on standard GCM model data; see (*27*, *31*–*34*)] may not sufficiently represent potential future TC changes, suggesting that further research on TC projections and regional-scale TC-produced ocean wave extremes (e.g., using regional high-resolution GCMs and regional synthetic TC events) is needed.

In conclusion, we highlight that present-day uncertainties in extreme wave height derived from the existing ensemble of global wave hindcasts/reanalysis exceed and/or are comparable to that of global wave model projections (and associated uncertainties). Hence, uncertainties inherent to both future projections and present-day estimates need to be accounted for and combined in comprehensive offshore and coastal risk assessments relying on extreme wave data. Otherwise, incorporating ongoing improvements in climate modeling without addressing uncertainties in the wave climate system may provide little benefit for many broad-scale impact and adaptation assessments.

## MATERIALS AND METHODS

### Contemporary global wave reanalysis and hindcasts

AMAX *H_s_* data were taken from the first coordinated multiproduct ensemble of global wave reanalysis/hindcast product (*37*). This recently assembled dataset was compiled under a standardized research framework (*37*) and provides general and extreme wave statistics (including AMAX *H_s_* calculated from sub-daily time series) for 14 global wave products produced using third-, fourth-, and fifth-generation atmospheric reanalyses as forcing (*37*). In this analysis, we use all the global wave products (two reanalysis and nine hindcasts) that cover the full temporal record length available of 35 years (between 1980 and 2014) (*37*). To further expand our sampling space, we also included a JRA55-forced global wave hindcast (KU-JRA55) that spans 32 years (between 1980 and 2012). The full description of all global wave product datasets (including their validation) is extensively provided elsewhere (*37*), and hence, we only provide a brief summary of their key characteristics along with respective acronyms (table S1).

### Global wave buoy measurements

In situ wave buoy stations with long-term data records are relatively scarce worldwide. Hence, wave buoy selection was based on a compromise between buoy availability and data suitability. We used time series of AMAX *H_s_* calculated from (hourly to 3-hourly) measurements extracted from all available wave buoy record networks that meet a number of previously adopted quality requirements (*33*) to ensure robust estimates and a suitable comparison against reanalysis and hindcasts across a maximum number of suitable locations. We used all buoy stations that are: (i) moored at water depths of less than 45 m to ensure that ocean waves are not heavily affected to shallow-water nonlinear processes unresolved by current global wave models; (ii) sufficiently far from land so that corresponding wave model output data are located at sea; and (iii) resultant time series of AMAX *H_s_* must comprise at least 20 values chosen on the basis of (*33*): (i) AMAX values for buoys sited above 40°N or below 40°S (extratropical region) are selected from all years with >60% of sub-daily data available during boreal (January, February, and December) and austral (June, July, and August) winter seasons (respectively), and (ii) AMAX values for buoys located below 40°N or above 40°S (subtropical and tropical regions) are selected from all years with >60% of sub-daily data available. In total, we assembled a network of 64 wave buoys, which we use to estimate $H_{s}^{n}$ (fig. S1 and table S2).

### Comparison against model estimates

The global wave model products used provide continuous time series of AMAX *H_s_* for 35 years (see "Contemporary global wave reanalysis and hindcasts" section). Nonetheless, our quality-controlled buoy records are often shorter (table S2). Therefore, to provide a coherent comparison between $H_{s}^{n}$ estimated from model and buoys, we removed any years from product time series of AM data that are missing within the observational records when comparing buoys and models (while always ensuring a 20-year record length) before calculating return periods.

### Statistical extreme value models

Applying EVA approaches to observed (or modeled) extreme *H_s_* data allows the quantification of return periods that are longer than available records (*38*, *39*). Now, there is an existing range of statistical EVA methods that can be used to derive $H_{s}^{n}$ (*38*). Although specific methods could be favored when certain criteria are fulfilled (e.g., sufficient data length and availability of sub-daily data), there is no universally accepted standard method for extreme wave analysis (*21*, *38*). Two reference extreme value distributions (GEV and GUM maxima) have been widely used to estimate $H_{s}^{n}$ from AMAX series (*33*, *34*) and are used here

$${H_{s}^{n}}_{\mathrm{GEV}-\mathrm{AMAX}}=\mu -\frac{\sigma }{k}\;\left(1-{\left\{-\log\left(1-\frac{1}{n}\right)\right\}}^{-k}\right)$$ (1)

where μ is the location parameter, σ is the scale parameter, and *k* is the shape parameter, with *k* > 1 representing a heavy-tailed distribution (Fréchet family) and *k* < 1 representing a bounded upper limit–tailed distribution (Weibull family). The GUM (maxima) distribution assumes that *k* = 0 and represents a two-parameter light tailed distribution

$${H_{s}^{n}}_{\mathrm{GUM}-\mathrm{AMAX}}=\mu -\sigma \;\log\left\{-\log\left(1-\frac{1}{n}\right)\right\}$$ (2)

In this analysis, we assumed a conservative approach and constrain estimates to 50-year return events ($H_{s}^{50}$) to prevent unreliable extrapolation periods. In all cases, the statistical distribution parameters were calculated using the asymptotically optimal maximum likelihood estimator. Alternative parameter estimation methods exist (e.g., method of moments or L-moments), but we have not considered them because their influence would be insignificant relative to the key uncertainties discussed.

### Suitability of underlying data and statistical model fit

To have a consistent comparison of the different uncertainties across the global ocean, the same extreme value distribution must be adopted (*33*, *66*). There is a range of accepted methods that can be used to assess how well extreme value models fit a given climate dataset. We compared the suitability and the fit of GEV-AMAX and GUM-AMAX using different methods.

#### Anderson-Darling statistical test

We used the Anderson-Darling test (*67*) at 5% confidence level to determine whether the time series of AMAX *H_s_* follow a GUM distribution (our null hypothesis) versus any other (extreme) distribution. The results show that the null hypothesis is not rejected (i.e., there is no significant departure from the GUM distribution) across more than ~85% of the global ocean regardless of the global wave model product used (fig. S13). The regions where the null hypothesis is rejected (*P* < 0.05) are limited to specific TC dominated as explained below.

#### Significance of shape parameter fit

We assessed the statistical significance of the fit of the GEV-AMAX shape parameter *k* at 95% confidence level using its estimated confidence limits. The results show that the fit of *k* is not statistically significant from zero across more than ~85% of the global ocean (fig. S9). The areas where *k* is found to provide a significant fit (less than 15%) (fig. S9) are specific TC-dominated areas, typically characterized by heavy-tailed distributions, where the suitability of the GUM distribution is compromised on the basis of the Anderson-Darling test (fig. S13). These results are consistent with previous global-scale analyses (*33*), which used GUM-AMAX after showing that such model provides an overall more suitable fit compared to GEV-AMAX when applied to global wave hindcast data (GOW2) and CMIP5-driven global wave simulations.

#### Akaike information criterion

To further support our results, we determined the Akaike information criterion (corrected for small samples) (AICc) (*68*) for both GUM-AMAX and GEV-AMAX. The AICc is an estimator of prediction error used across different scientific research fields (*69*, *70*), which asymptotically selects the extreme value distribution that minimizes the mean squared errors of the estimation. The AIC corrected for small sample sizes (*N*/*W* < 40) is provided by

$$\mathrm{AICc}=-2\log L(\hat{\theta })+2W+\frac{2W(W+1)}{N-W-1}$$ (3)

with $L(\hat{\theta })$ representing the maximized log-likelihood, *W* representing the number of estimated parameters used to achieve that log-likelihood, and *N* representing the sample size. The AICc is calculated on the basis of a compromise between goodness of fit and model complexity with lower AICc values indicating a better-fit model. The results are provided in fig. S14.

#### Bayesian information criterion

We also determined the Bayesian information criterion (BIC)

$$\mathrm{BIC}=-2\log L(\hat{\theta })+W\log(N)$$ (4)

which selects the model that maximizes the posterior model probability. The comparison of the AICc and BIC values for GUM-AMAX and GEV-AMAX shows that GUM-AMAX provides a better-fit model across 70 to 78% and 78 to 86% of the global ocean, respectively (depending on the global wave product used) (figs. S14 and S15). The only areas where GEV-AMAX lends a better-fit model correspond to specific TC-dominated regions (less than 15 to 20%) where GUM-AMAX could result in an underestimation of heavy-tailed distributions (and associated estimates). These results (combined with the fact that our ensemble projections of *k* exhibit no robust or statistically significance changes over most of the global ocean) support using GUM-AMAX as reference in this analysis, consistent with previous research (*33*).

### Clustering methodology

We used an agglomerative hierarchical clustering analysis, with the similarity criterion defined by Ward’s analysis of variance (ANOVA)–based minimum variance algorithm (*71*). The clustering method was used without imposing any restrictions on the number and size or any a priori assumptions of clusters. The initial cluster distances (used within the Ward’s minimum variance method) were obtained using a multidimensional approach, where the pairwise Euclidean distance (*D*) among model products is derived at every grid node, rather than spatially averaged, thus clustering products with high similarity regarding spatial pattern and magnitude (*26*)

$$D_{i,j,k}=\sqrt{\sum_{k=1}^{w}{\left(x_{i,k}-x_{j,k}\right)}^{2}}$$ (5)

where *x*_*i*,*k*_ and *x*_*j*,*k*_ are the $H_{s}^{50}$ estimates derived from two given global wave products *i* and *j*, respectively, at grid point *k* with *w* equal to the number of ocean grid nodes. We tested alternative clustering distance metrics and obtained consistent results.

### Weighted ensemble mean of global wave hindcasts and reanalyses

The findings of our skill analysis (figs. S2 and S3), along with the sparsity of global wave buoys records (fig. S1), preclude weighting individual ensemble models based on their relative skill. Nevertheless, we show that present-day $H_{s}^{n}$ estimates are strongly dependent on reanalysis forcing (fig. S3), and therefore, a weighted multiproduct ensemble mean was calculated by applying weighting factor to each global wave product

$${\bar{H_{s}^{n}}}_{k}=\frac{\sum_{v=1}^{12}({H_{s}^{n}}_{v,k}\times W_{v,k})}{\sum_{v=1}^{12}W_{v,k}}$$ (6)

where ${H_{s}^{n}}_{v,k}$ represents the estimate of $H_{s}^{n}$ according to the ensemble model product *v* at each grid point *k* and *WT*_*j*,*k*_ its weighting factor representing the number of ensemble products with that same atmospheric reanalysis forcing among all products available.

### Global wave model projections

Time series of AMAX *H_s_* were taken from the largest ensemble of CMIP5-based global ocean wave projections available to date (spanning different global climate model forcings and global wave downscaling methods) (table S3). This ensemble and its members have been extensively described and validated (*26*, *37*). The data were extracted over two available representative time slices: a reference historical period (1980 to 2005) and a future climate period (2080 to 2100) that assumes a high-end warming scenario (RCP8.5). In total, AMAX *H_s_* extracted from 38 simulations were used to estimate projected future changes and associated uncertainties.

### Calculation of projected future changes

The projected relative change in $H_{s}^{n}$ was calculated as percentage change (and absolute change) for each ensemble model member following (*26*)

$$\Delta {H_{s}^{n}}_{k}=\frac{\left({H_{s}^{n}}_{j,k}^{\mathrm{Future}}-{H_{s}^{n}}_{j,k}^{\mathrm{Present}}\right)}{{H_{s}^{n}}_{j,k}^{\mathrm{Present}}}$$ (7)

where $\Delta H_{s\;j,k}^{n}$ represents the projected future changes according to the ensemble member *j* at each grid point *k*. We determined a weighted multimember ensemble mean of projected future change by applying weighting factors to each ensemble member (table S3) on the basis that projected changes are strongly dependent on climate model forcing (*26*, *72*)

$${\bar{\Delta H_{s}^{n}}}_{k}=\frac{\sum_{j=1}^{39}(\Delta {H_{s}^{n}}_{j,k}\times W_{j,k})}{\sum_{j=1}^{39}W_{j,k}}$$ (8)

where *W*_*j*,*k*_ is the weighting factor for the ensemble model member *j*, calculated as the number of ensemble members with that same climate model forcing among all members available.

### Calculation of uncertainty

#### Present-day uncertainty

The uncertainty associated with using different global wave reanalysis/hindcast products (here, present-day uncertainty) is calculated using the IQR of the entire ensemble estimates of $H_{s}^{n}$ (Fig. 4B). The IQR is considered to be the most suitable measure of variability for nonnormal or skewed data distributions and/or datasets with outliers. We also calculate the max-min difference between ensemble estimates, and the results are consistent (fig. S6).

#### Projection uncertainty

For consistency, the uncertainty related to global extreme wave projections (future uncertainty) is calculated using the IQR of the projected change estimates in $H_{s}^{n}$ from the different members. In this case, we apply a bootstrapping procedure to the IQR values (i.e., we subsampled 12 ensemble members at the time, for 1000 times, and calculated the mean of the 1000 IQR values) to match the number of samples when comparing the present-day and future uncertainties. The results are consistent with those obtained without applying any bootstrapping.

#### Combined uncertainty

The different uncertainties discussed (that is, IQRs associated with contemporary $H_{s}^{n}$ estimates and those associated with global projections of extreme waves) are here combined using the root of the sum of their respective IQR values as follows

$${\mathrm{Combined}}_{\mathrm{IQR}}=\sqrt{\sum_{j}^{N=2}{\left({\mathrm{IQR}}_{j}\right)}^{2}}$$ (9)

where *N* is the number of individual uncertainties (here, *N* = 2) that are being combined.

### Offshore and coastal infrastructure data

The exact locations of the offshore wind farm projects were extracted from a publicly available global remote sensing–based offshore wind turbine database obtained from Sentinel-1 synthetic aperture radar extensively validated time-series images (*73*). Because the dataset contains all the existing wind turbines deployed, we considered each aggregation of turbines as a single farm project based on their central coordinates (*73*). The locations of the global deep offshore oil and natural gas platforms were extracted from a global dataset that was published as part of a global analysis of the projected footprint of marine-built structures (*9*). The global open-coast seaports were obtained from a published global-scale analysis (*74*) based on a refined version of the World Port Index database (provided by the National Geospatial-Intelligence Agency).

## Supplementary Materials

### This PDF file includes:

Supplementary Information Figs. S1 to S16 Tables S1 and S2 References
