# Supplementary material for: Understanding uncertainties in contemporary and future extreme wave events for broad-scale impact and adaptation planning
Source: Sci Adv. 2023 Jan 11;9(2):eade3170. doi: 10.1126/sciadv.ade3170 (PMC9833663; doi:10.1126/sciadv.ade3170)
Supplement: Supplementary file 1 — Supplementary Information Figs. S1 to S16 Tables S1 and S2 References [file sciadv.ade3170_sm.pdf]

Science Advances
AAAS

Supplementary Materials for

Understanding uncertainties in contemporary and future extreme wave events for broad-scale impact and adaptation planning

Joao Morim *et al.*

Corresponding author: Joao Morim, jmorimnascimento@ucf.edu

Sci. Adv. **9**, eade3170 (2023)
DOI: 10.1126/sciadv.ade3170

This PDF file includes:

Supplementary Information
Figs. S1 to S16
Tables S1 and S2
References

Supplementary Information

Global wave model products

In this section, we provide a brief description of the global wave products (*37*), developed at different research centres, with the details and differences of each contribution provided within Supplementary Table S1.

Global wave hindcasts

NCEP/NCAR-based products

IHC-GOW1.0: Reguero et al.[75] developed the Global Ocean Waves (GOW1.0) wave hindcast by forcing the WaveWatch III (hereafter WW3) global wave model version 2.22, with 6-hourly surface wind fields obtained from the NCEP/NCAR atmospheric reanalysis and hourly sea-ice forcing fields from the MOM3 sea-ice model. The wave model was implemented using default ST2 source-term wave physics[24], with the wave spectra discretised over 25 frequencies and 72 directions. The model was implemented on a global grid with 1.5° × 1.0° spatial resolution with wave model outputs available at hourly intervals. GOW1.0 global wave hindcast has undergone a series of calibration and validation methods against H_s measurements extracted from satellite altimeters and buoy instruments (*37*).

NCEP CFSR-driven products

CSIRO-CFSR: Smith et al.[76] developed a global wave hindcast using versions v4.08/v4.18 of the WW3 wave model. The atmospheric forcing of the WW3 model were hourly surface winds derived from the CFSR atmospheric reanalysis. Sea-ice concentration fields at hourly intervals from the MOM4 sea-ice model were used as forcing. The model was setup at 1° resolution (up to 0.4° resolution across the Australian and Central Pacific regions) using ST4[24] source-term physics with default settings. The model wave spectra are discretised across 29 frequencies and 24 directions with model outputs available at hourly resolution. The CSIRO-CFSR global wave hindcast has been extensively validated against available satellite altimeter data and against the Australian national buoy network and a selection of NDBC-operated wave buoys (*37*).

IHC-GOW2.0: Perez et al.[24] produced an updated global wave hindcast of GOW1.0[SM1] driven by hourly surface wind fields from the CFSR atmospheric reanalysis and hourly sea-ice forcing from the MOM4 sea-ice model. GOW2.0 is based on version 4.18 of the WW3 and uses default ST436 source-term wave physics[24] on a multi-grid scheme with a series of two-way nested grid domains, covering the global ocean basins and continental shelf areas at ~0.5° and ~0.25° spatial resolution, respectively. The wave spectra are discretised over 32 frequencies and 24 directions and wave model outputs are available at 1-hourly intervals. The model data has been validated against wave spectral information from wave buoy stations and multimission satellite altimeter measurements (*37*).

JRC-CFSR: Mentaschi et al.[28] developed a global wave hindcast product by forcing the WW3 wave model version v4.08 with 1-hourly surface wind fields from the CFSR global atmospheric reanalysis. The WW3 wave model was implemented without sea-ice forcing. The model setup uses ST4 source-term physics[24] with the wave growth parameter (β_{max}) adjusted to 1.52. The model domain consists of a global grid at 1.5° spatial resolution, with multiple nested sub-grids implemented across different basins using 0.5° and 0.25° spatial resolutions. Model outputs are available at 3-hourly resolution with no validation reported (*37*).

IFREMER-CFSRSAT: Stopa et al.[78] created a global wave hindcast product by forcing WW3 model version 5.16 with satellite-adjusted surface winds from the CFSR atmospheric reanalysis and hourly sea-ice forcing obtained from the MOM4 sea-ice model. The model was setup using ST4[24] source-term physics with β_{max} adjusted to 1.30 and wave spectra discretized across 24 frequencies and 32 directions. The model wave outputs are archived at hourly resolution at 0.5° spatial resolution. The IFREMER-CFSRSAT was extensively validated using multiple NDBC-based wave buoy and microseism data (*37*).

ECMWF ERAI-driven products

JRC-ERAI: Mentaschi et al.[28] generated a global wave hindcast by forcing WW3 wave model version 4.08 with 6-hourly surface winds from ECMWF ERA-Interim atmospheric reanalysis.

The WW3 model was run without sea-ice forcing fields, using the ST4 source-term physics[24] with default model settings. The WW3 wave model was implemented at 1° spatial resolution, with wave outputs available at 12-hourly intervals. No validation is reported for the JRC-ERAI (*37*).

NOC-ERAI: Bricheno and Wolf[29] developed a global wave hindcast using WW3 wave model version 3.14 forced by 6-hourly surface winds derived from ECMEF ERA-Interim atmospheric reanalysis and daily sea-ice fields from the LIM2 sea-ice model. The model was implemented using default ST2 source-term physics[24] with wave spectra discretized across 30 frequencies and 36 directions. The spatial resolution was set at 0.7° × 0.5° with outputs available at 1-hourly intervals (*37*).

ECMWF ERA5-driven products

ECMWF-ERA5H: ECMWF-ERA5H: Bidlot et al.[79-80] created a global wave hindcast product by forcing EC-WAM wave model with 1-hourly surface winds and sea ice cover from ECMWF ERA5 atmospheric reanalysis. The EC-WAM wave model globally implemented globally at 0.5° spatial resolution, with model wave spectral ordinates discretized over 36 frequencies and 36 directions. The model settings included ST4 source-term physics[24] tuned to ECMWF Earth System model[79,80]. The ECMWF-ERA5H have been compared against both satellite altimeter measurements and wave buoy observations (*37*).

JMA JRA-55-driven products

KU-JRA: Shimura et al.[81] produced a global wave hindcast by forcing the WW3 wave model version 4.18 with 6-hourly surface winds from the JRA-55 atmospheric reanalysis and monthly sea-ice concentration fields from the COBE sea-ice model. The wave model was implemented using the default ST4 source-term physics[24] with wave spectra discretized over 29 frequencies and 30 directions. The domain consists of a global grid with 0.56° spatial resolution and model outputs are archived at hourly intervals. The KU-JRA has been conducted against satellite and regional buoy data (*37*).

NASA MERRA2-driven products

IORAS-MERRA2: Sharmar et al.[25] generated a global wave hindcast by forcing WW3 wave model version v5.03 with 6-hourly surface winds obtained from the NASA GMAO MERRA2 atmospheric reanalysis. The wave model sea-ice concentration fields at hourly resolution were obtained from the MERRA2 coupled sea-ice model. The model was implemented using ST4[24] source-term physics with default settings, with wave model outputs archived on a global grid with 0.5° × 0.625° spatial resolution at 6-hourly intervals. The IORAS-MERRA2 global wave hindcast has been compared against visual observations (VOS) and satellite-retrieved altimeter measurements (*37*).

Global wave reanalyses

ECMWF-ERAI: Dee et al.[82] created the 4th generation of ECMWF´s atmospheric reanalysis by combining model data with historical observations. ECMWF-ERAI was generated using a 4D-VAR data assimilation system that is part of ECMWF Integrated Forecasting System (IFS) CY31R245 and provides 6-hourly atmospheric fields at 0.70° spatial resolution, globally. The wave parameters are available 3-hourly at 1.5° spatial resolution and are derived from a fully-coupled atmosphere-wave model (WAM) that describes the time-evolution of the wave spectra with assimilated satellite-retrieved wave height data (from 1991 onwards) to adjust model wave spectra based on assumptions about contributions of wind-sea and swell. ECMWF-ERAI wave parameters have been extensively compared against satellite altimetry and buoy records (*37*).

ECMWF-ERA5: Hersbach et al.[83] is the fifth generation of ECMWF atmospheric reanalysis which combines model data with vast amounts of past re-processed observations into a globally complete and consistent dataset. ECMWF-ERA5 was designed using a 4D-VAR sophisticated assimilation approach that is part of ECMWF Integrated Forecasting System (IFS) CY41R245 and provides 1-hourly atmospheric fields at 0.25° spatial resolution (from 1979-onwards). The model wave outputs are obtained using a fully-coupled atmosphere-wave model (WAM) using assimilated satellite radar altimeter-derived wave height data (from 1991-onwards). The model comprises various enhancements over its previous versions (ERA-I) with hourly model outputs available at 0.5° spatial resolution (*37*).

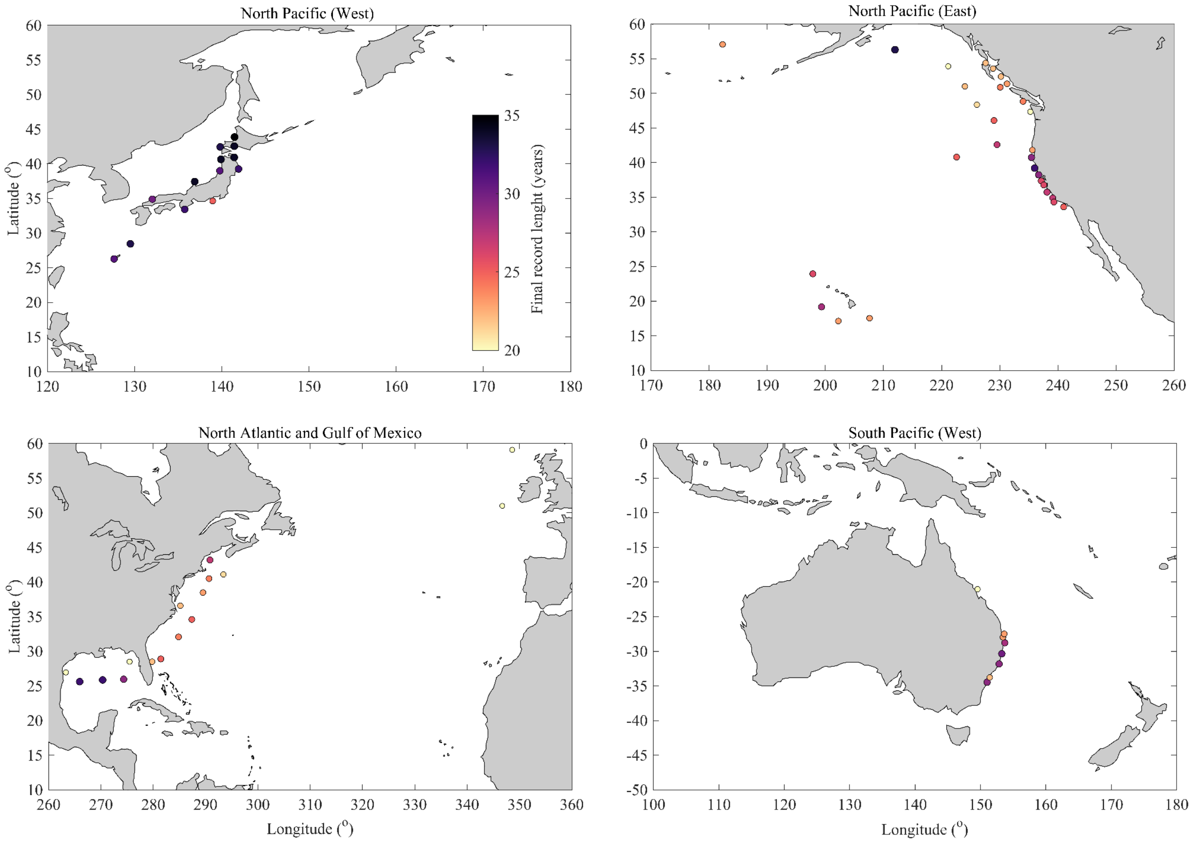

Supplementary Fig. S1 Geographical location and respective length of observational wave buoy records after applying selection criteria (Methods) (Supplementary Table S1).

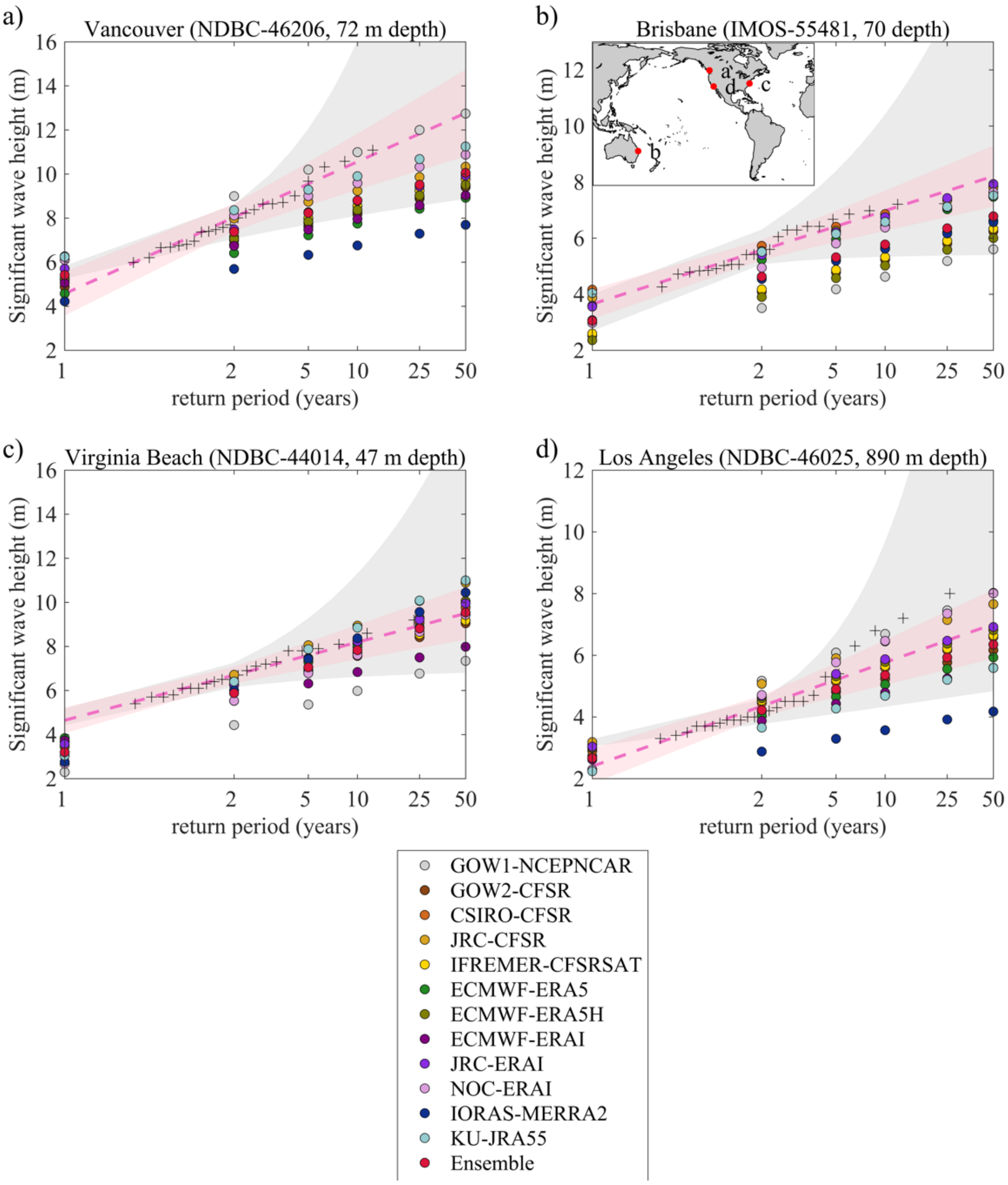

Supplementary Fig. S2 Return period significant wave heights (H_s^n) for representative wave buoy sites near major coastal cities. **a-f**, Plotting positions ('+') were derived from the observed annual maxima for each location and are therefore directly comparable to the GUM-AMAX fits from the different global wind-wave products (circles as per legend). Shaded grey bands represent the 95% confidence intervals of GUM-AMAX applied to the observations (and purple shaded bans represent the 95% confidence intervals of the GEV-AMAX) and the dashed line is the GUM-AMAX central estimate. The ID code for each buoy station is provided within each sub-panel (Supplementary Table S1).

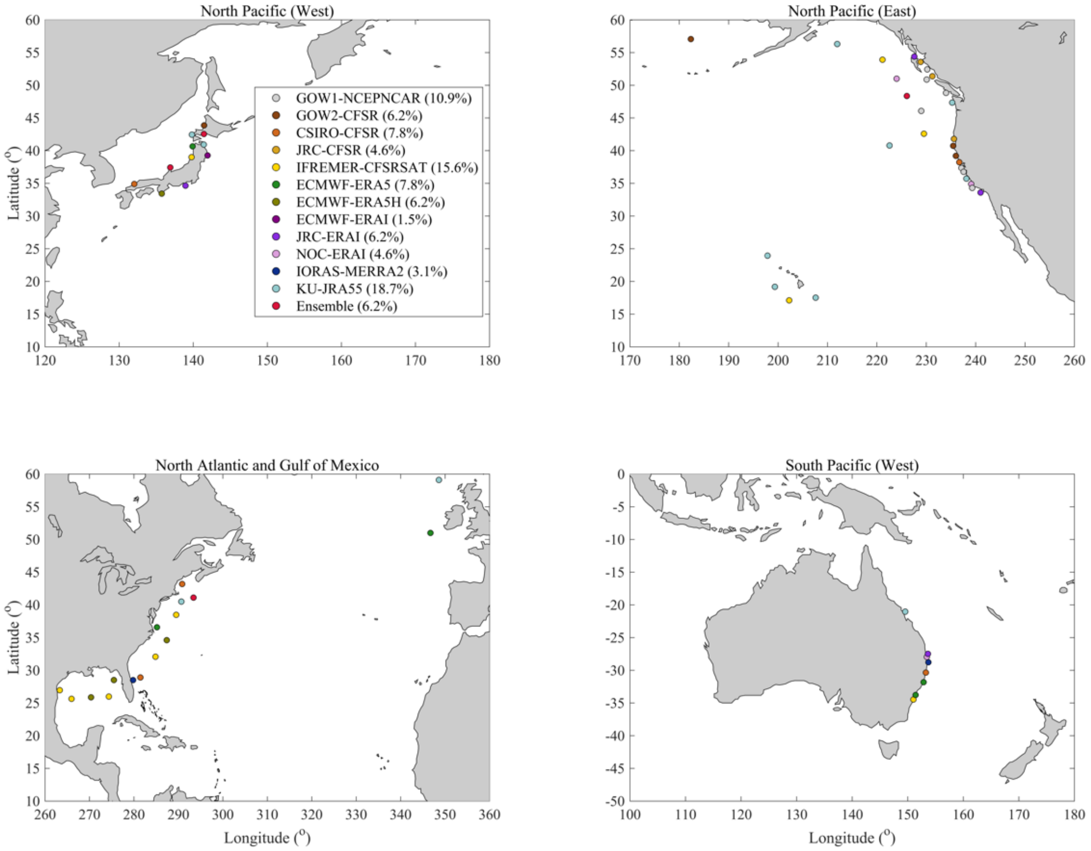

Supplementary Fig. S3 Global wave model products leading to lowest errors relative to observational records for 50-year return significant wave height (H_s^{50}). The percentage of sites where each global wave product leads to lowest relative errors is also shown (legend), respectively.

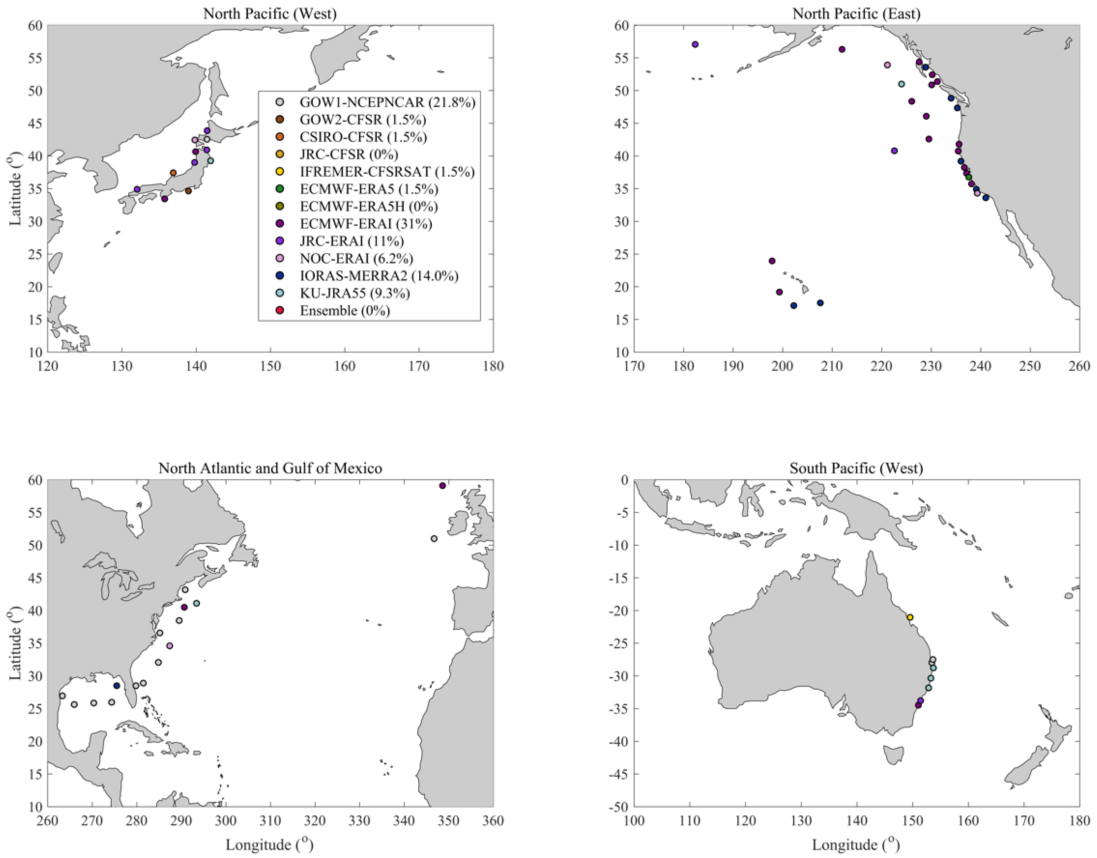

Supplementary Fig. S4 Global wave model products leading to highest errors relative to observational records for 50-year return significant wave height (H_s^{50}). The percentage of sites where each global wave product leads to highest relative errors is also shown (legend), respectively.

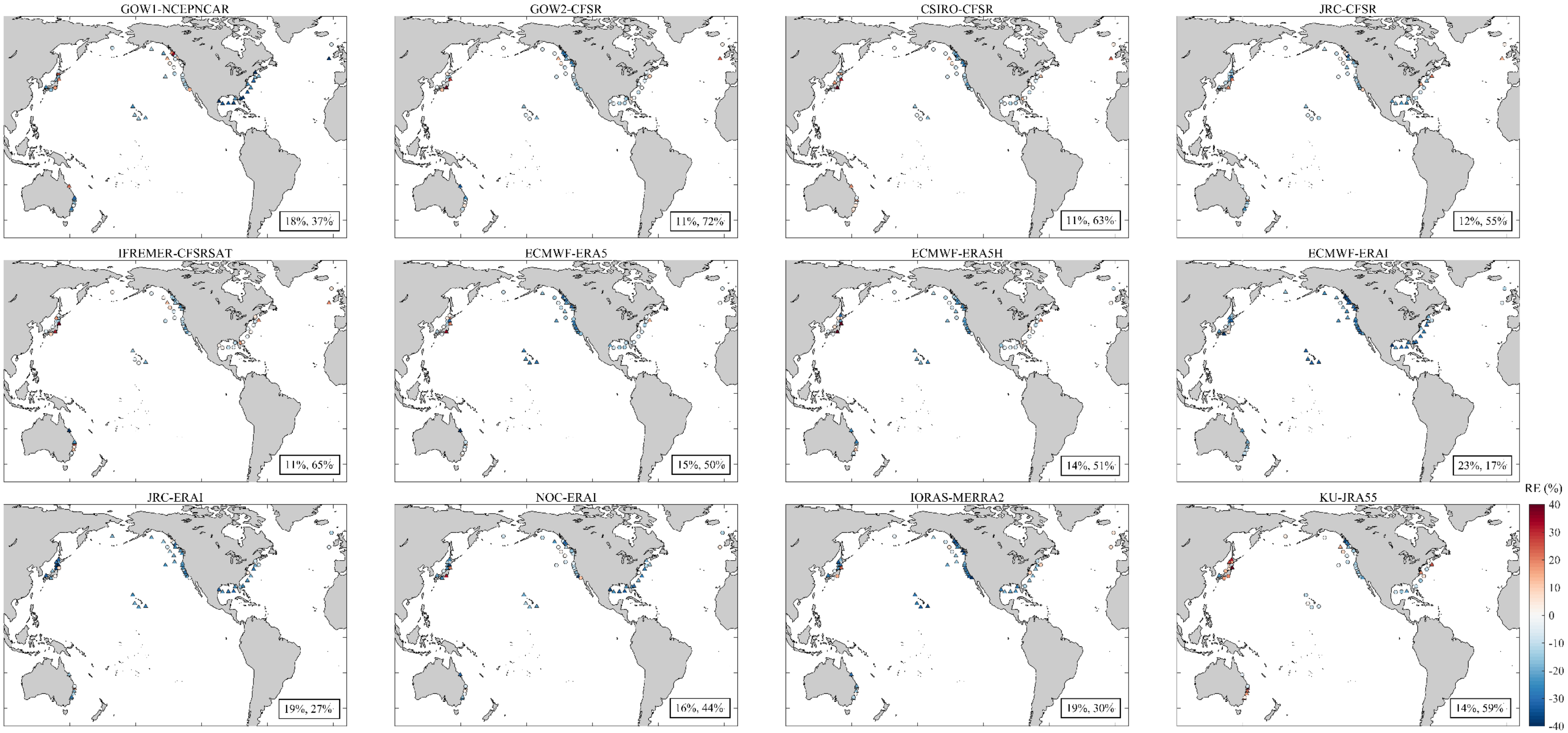

Supplementary Fig. S5 Error associated with 50-year return significant wave height (H_s^{50}) relative to observational wave records for each global wave product (Supplementary Table S1). The absolute error and percentage of sites that estimates fall within confidence levels of observations averaged across locations is presented each product within their respective panels., respectively. The triangle symbols represent the wave buoy locations where the central estimates from the model products do not fall within the confidence limits of the observations.

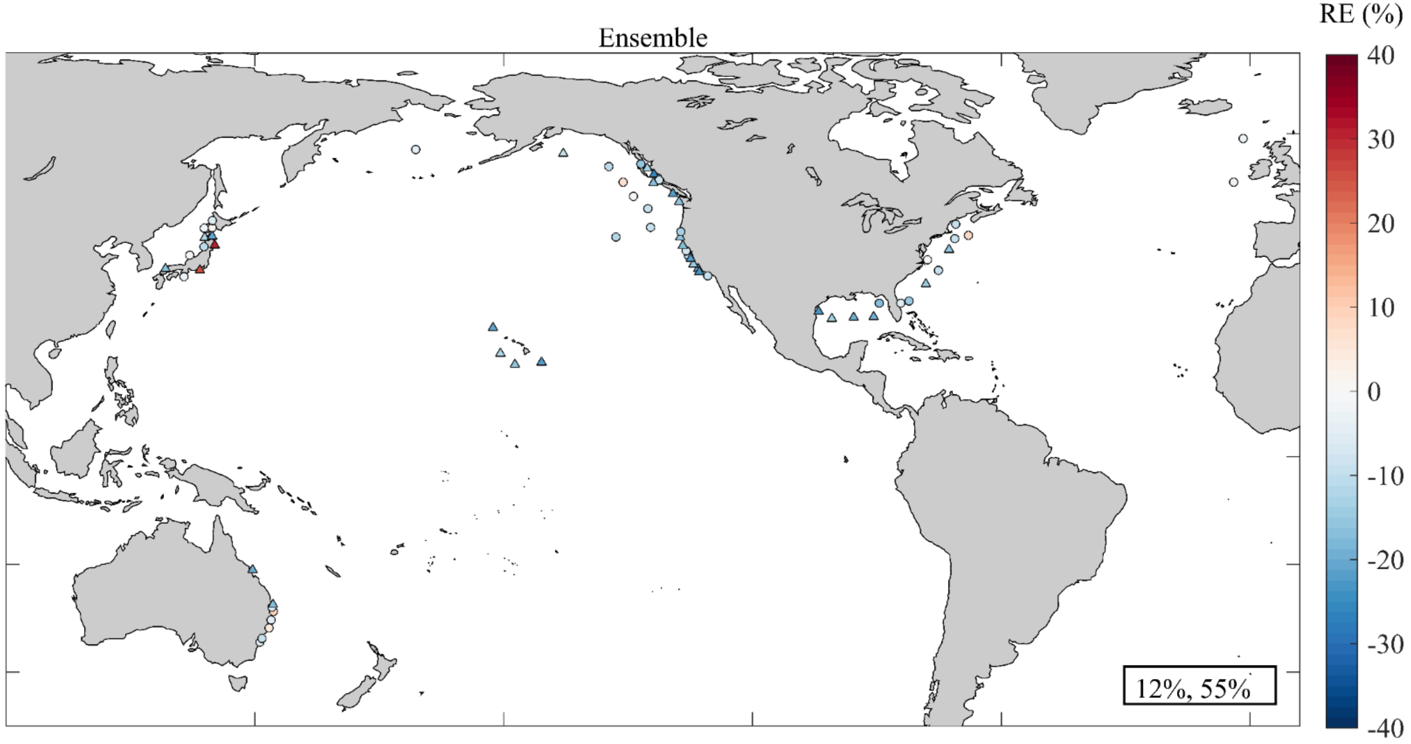

Supplementary Fig. S6 Error associated with 50-year return significant wave height (H_s^{50}) relative to observational wave records for weighted multi-product ensemble mean. The absolute error and percentage of sites that estimates fall within confidence levels of observations (GUM-AMAX) averaged across locations is presented, respectively. The triangle symbols represent the wave buoy locations where the central estimates from the model products do not fall within the confidence limits of the observations.

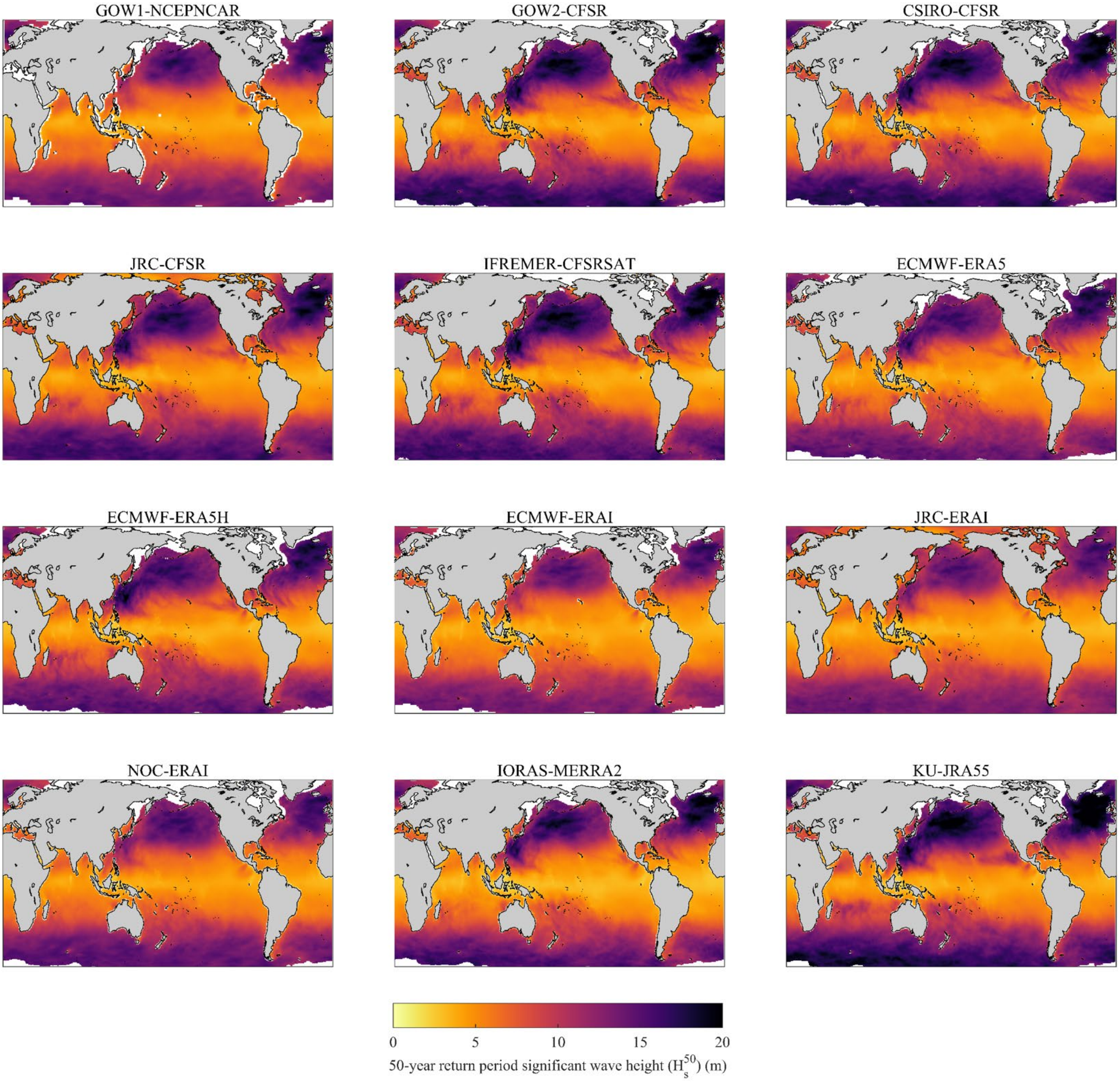

Supplementary Fig. S7 Estimates of 50-year return significant wave height (H_s^{50}) for the different global wave model products.

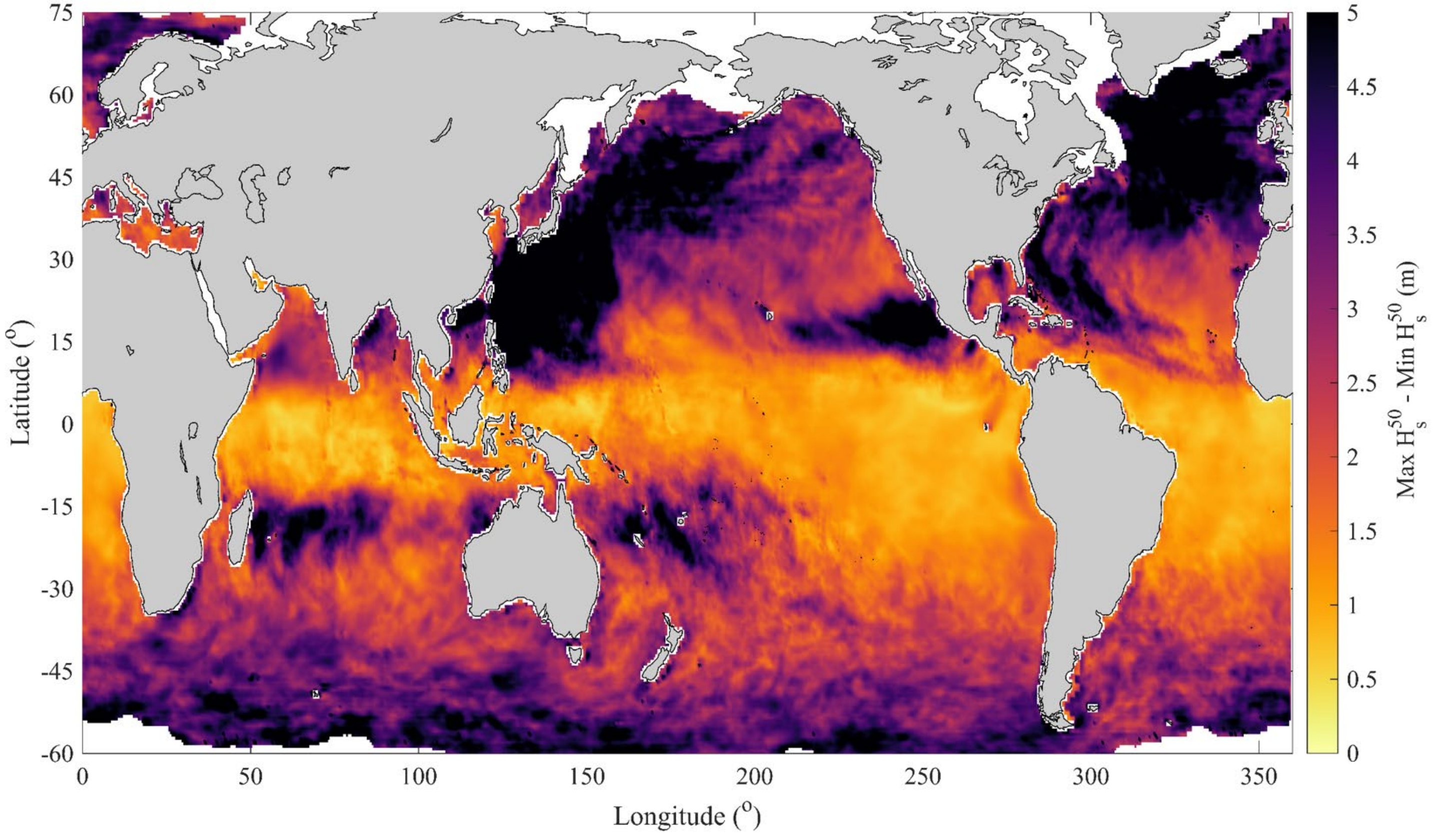

Supplementary Fig. S8 Maximum difference between ensemble estimates for 50-year return significant wave height across the global ocean.

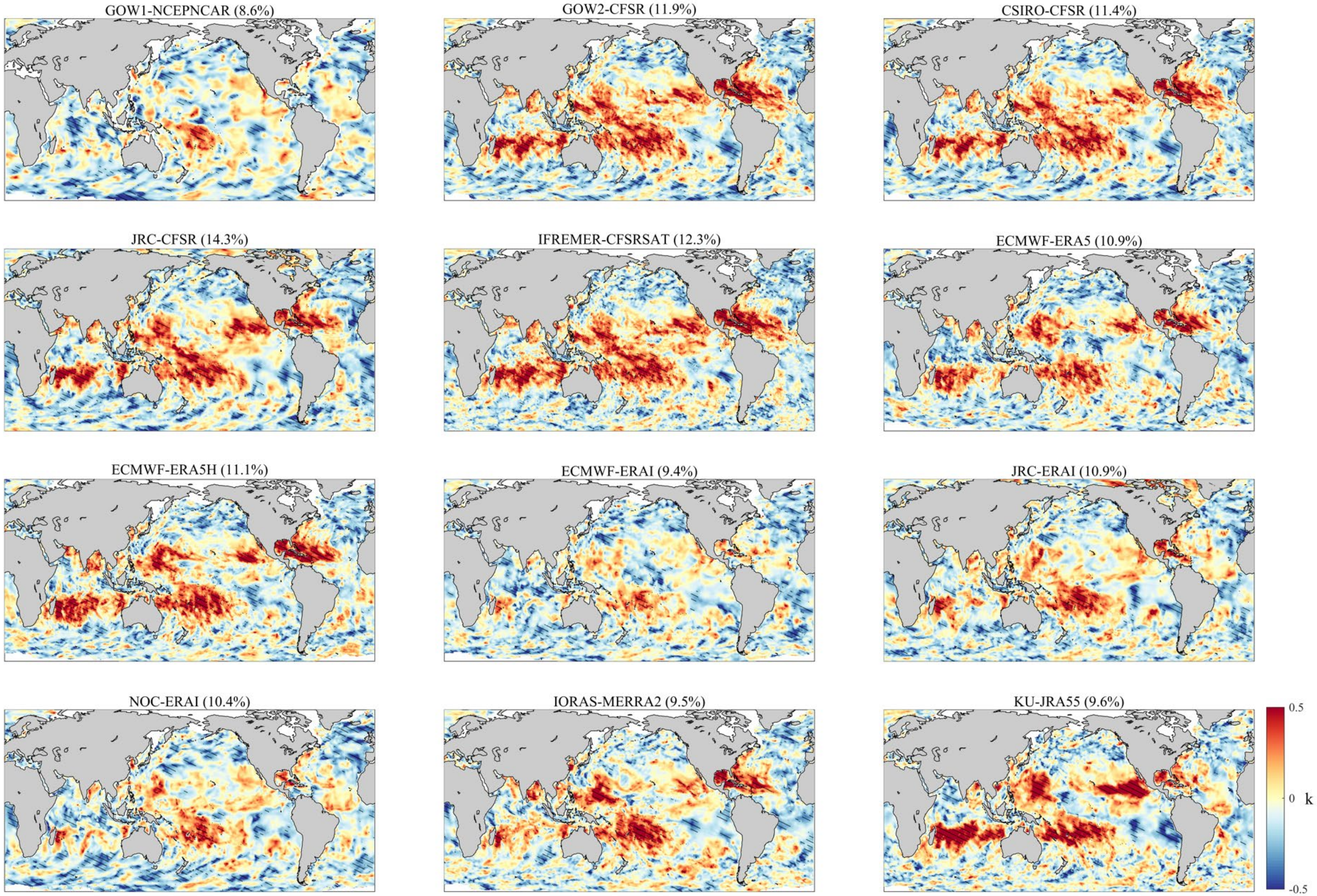

Supplementary Fig. S9 Shape parameter (*k*) and statistical significance of the shape fit for each global wave product. The percentage of global ocean area for which the shape value k shows a statistically significant fit (shaded regions) is shown on top of each panel.

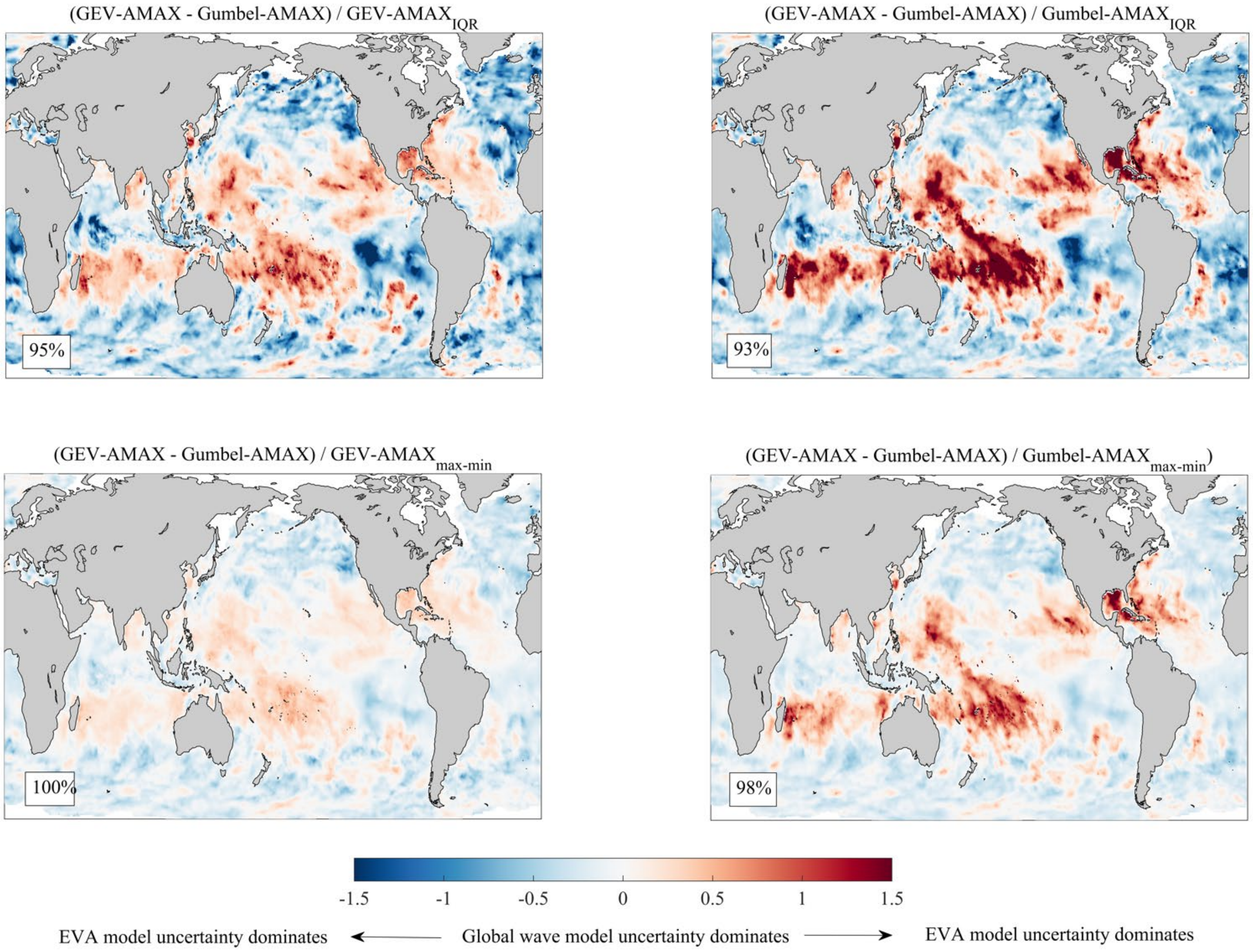

Supplementary Fig. S10 Relative importance of global wave model uncertainty and EVA statistical model uncertainty. Left-upper panel, Ratio of H_s^{50} (using GEV-AMAX method) minus H_s^{50} (using GUM-AMAX method) over interquartile range value (IQR) of H_s^{50} estimates from global wave products (GEV-AMAX). **Right-upper panel,** Same as (**a**), but using IQR of H_s^{50} estimates from global wave products using GUM-AMAX. **Left-upper and lower panels,** Same as first two panels but using difference between max-min H_s^{50} estimates instead of IQR. In all sub-panels, values represent weighted multi-member mean ratios, respectively. Note that EVA uncertainty only dominates when ratio is > 1 or < -1.

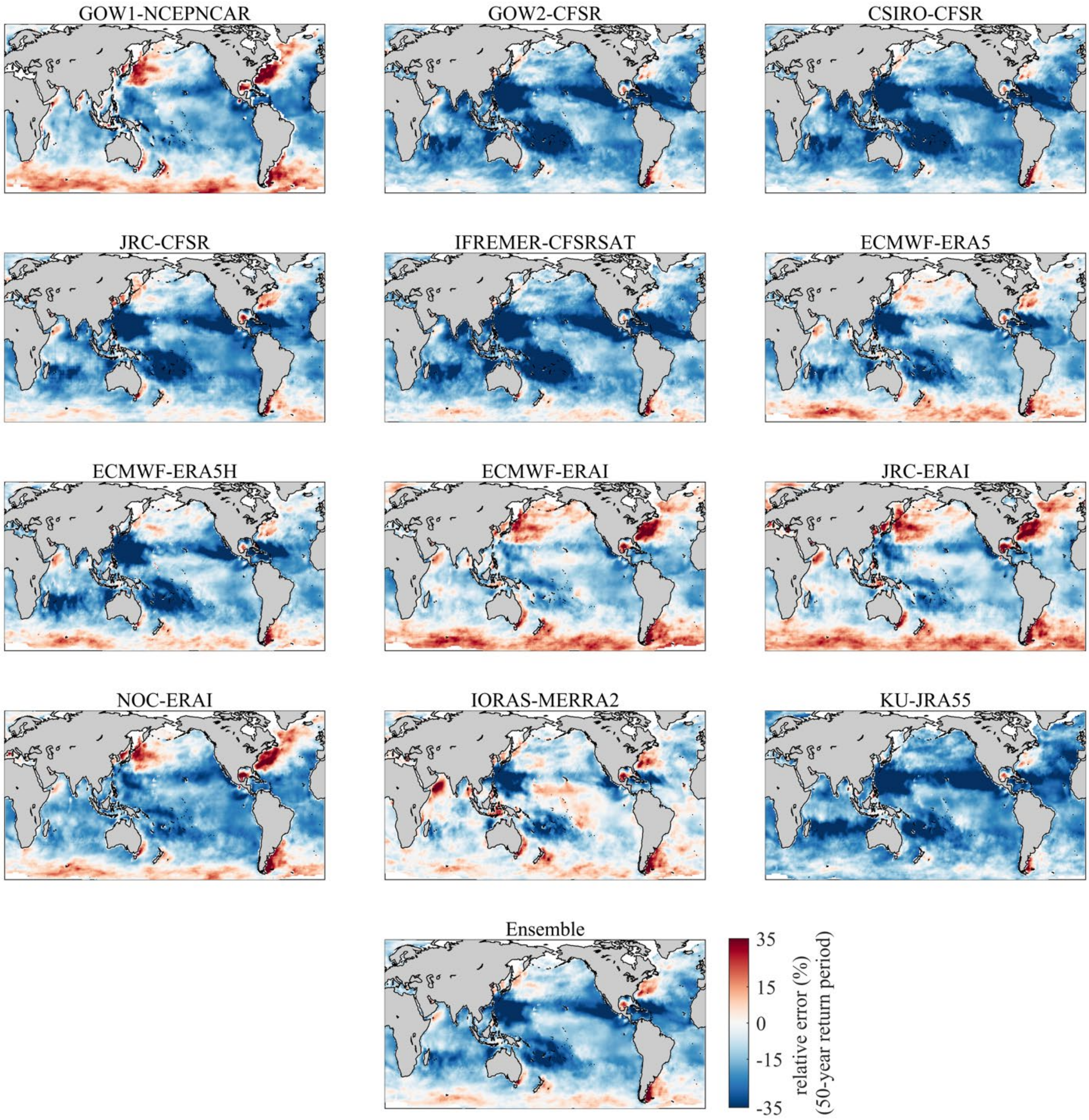

Supplementary Fig. S11 Error (%) in 50-year return significant wave height (H_s^{50}) for weighted ensemble mean relative to the different global wave products (see Methods). The weighted ensemble mean is computed for the historical time-slice (1980-2005).

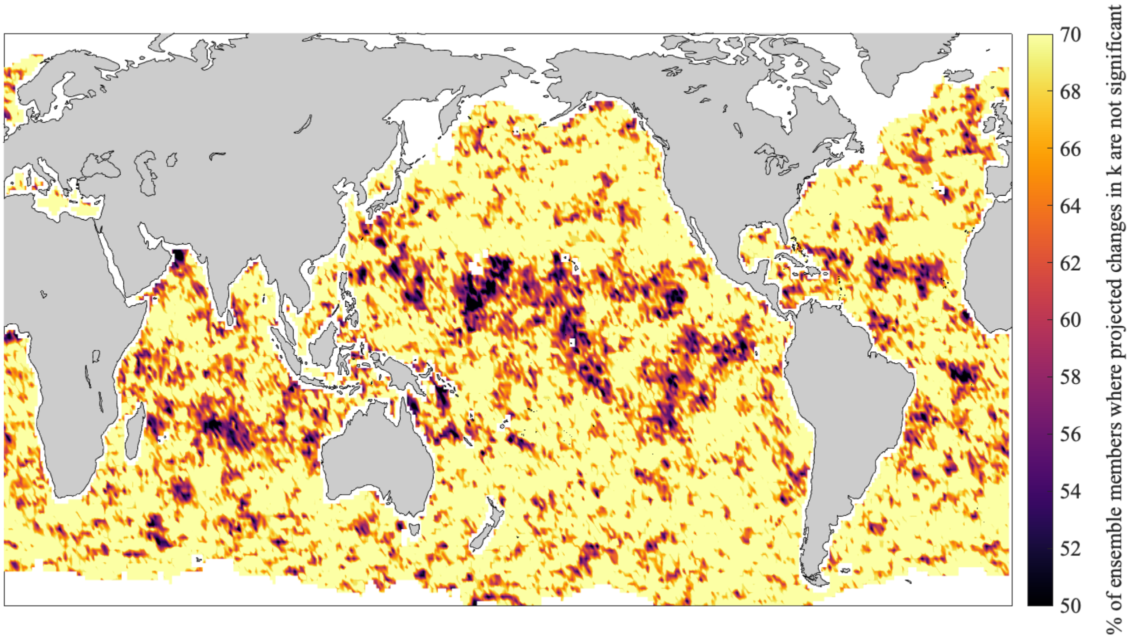

Supplementary Fig. S12 Percentage of ensemble members showing a statistically significant projected change in GEV-AMAX shape parameter (*k*) at 95% confidence level. At least 60% of ensemble members do not show significant changes in k across 91% of global ocean and 50% of ensemble members do not show significant changes in k across 98% of global ocean. There are no locations where our ensemble shows robust projected changes in k (i.e., statistically significant changes at 95% confidence level in at least 50% of members and an agreement in sign of change in more than 80% of significant members).

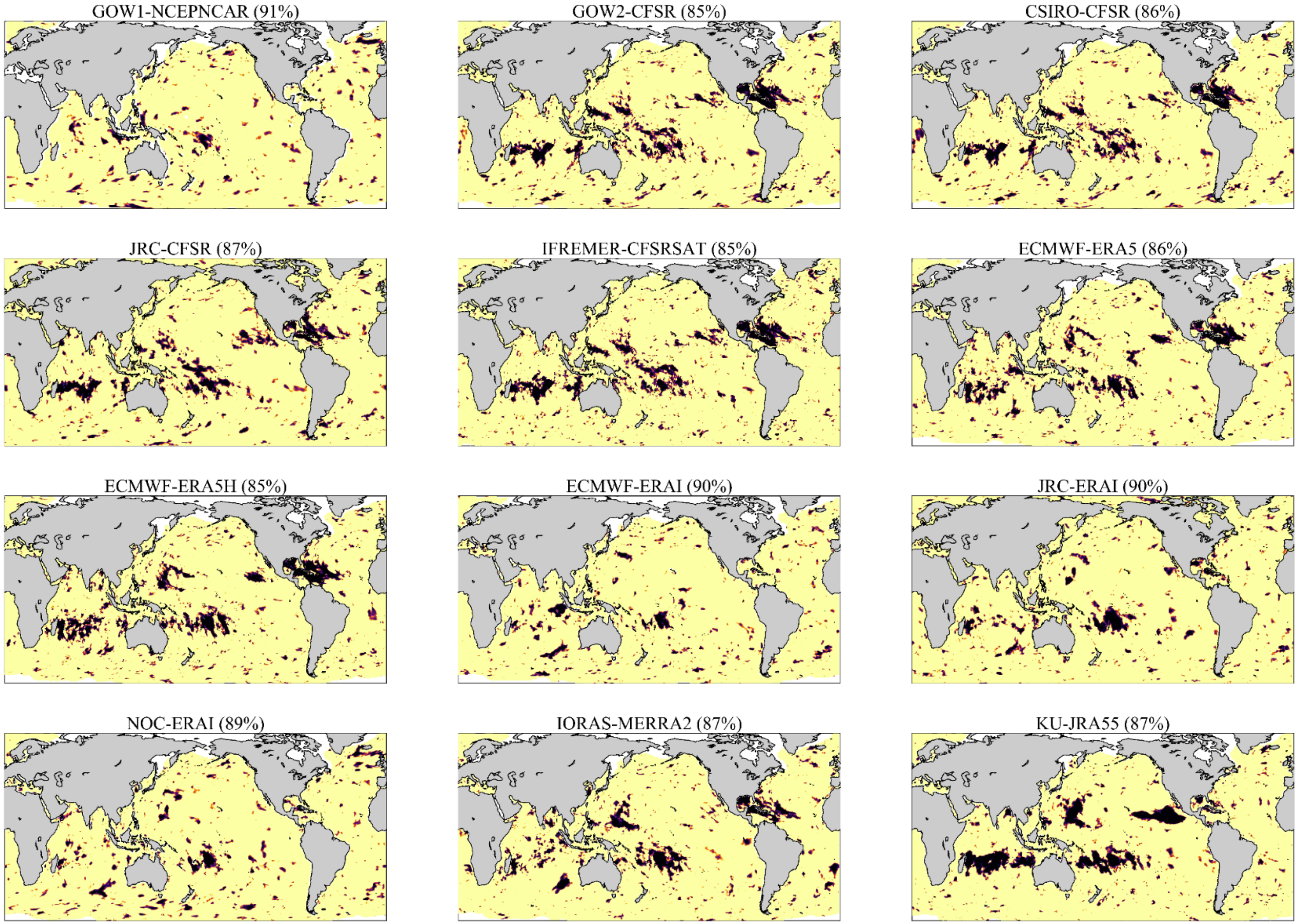

Supplementary Fig. S13 Results of Anderson-Darling statistical test for each global wave product used. Yellow represents regions where the null hypothesis ($p > 0.05$) is not rejected. The percentage of global ocean area for which the statistical test does not reject the null hypothesis ($p>0.05$) is shown on top of each panel.

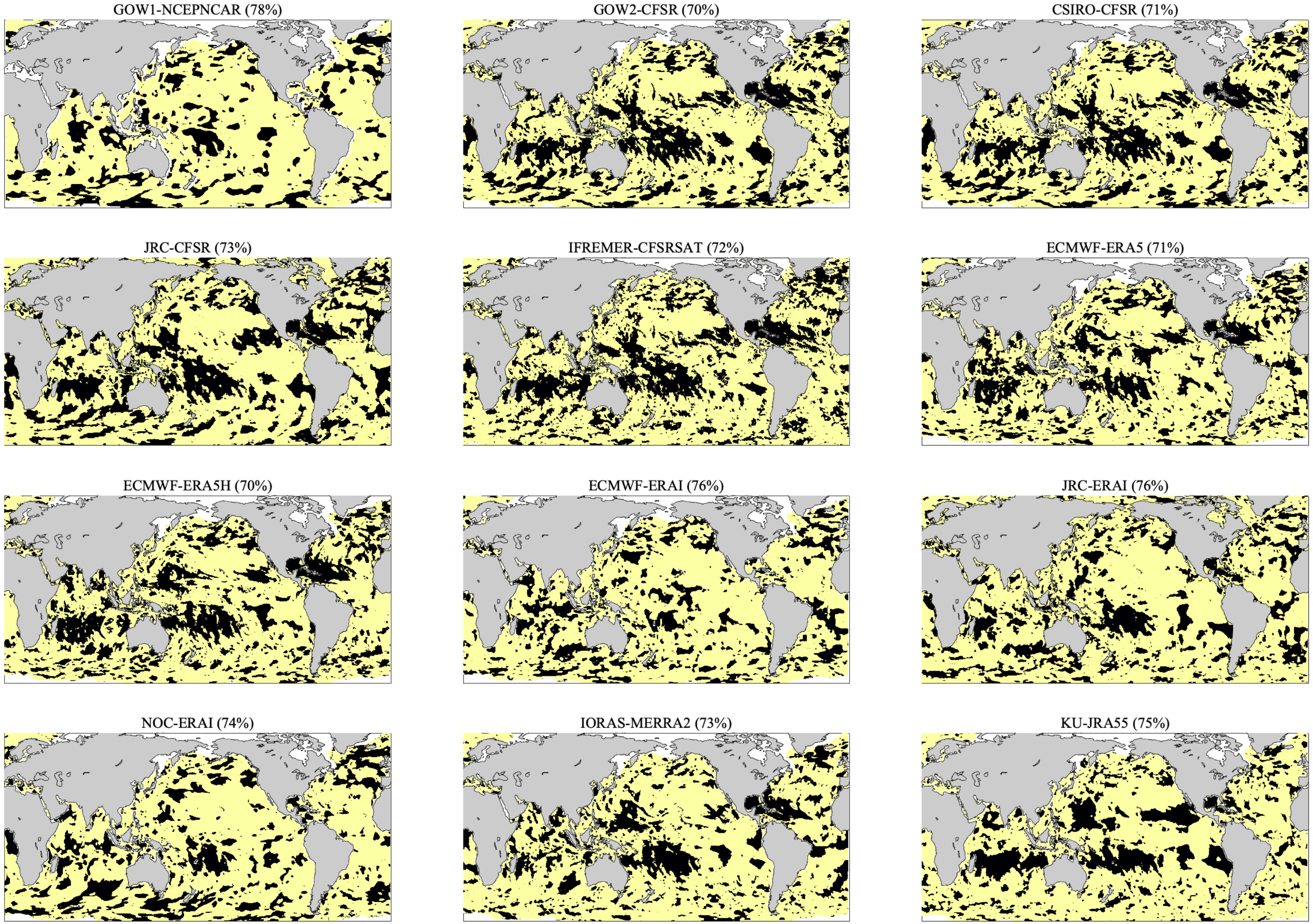

Supplementary Fig. S14 Results of AICc criterion (Methods). Yellow represents regions where AICc (GEV-AMAX) > AICc (GUM-AMAX). The percentage of global ocean area for which the AICc (GEV-AMAX) > AICc (GUM-AMAX) is shown on top of each panel.

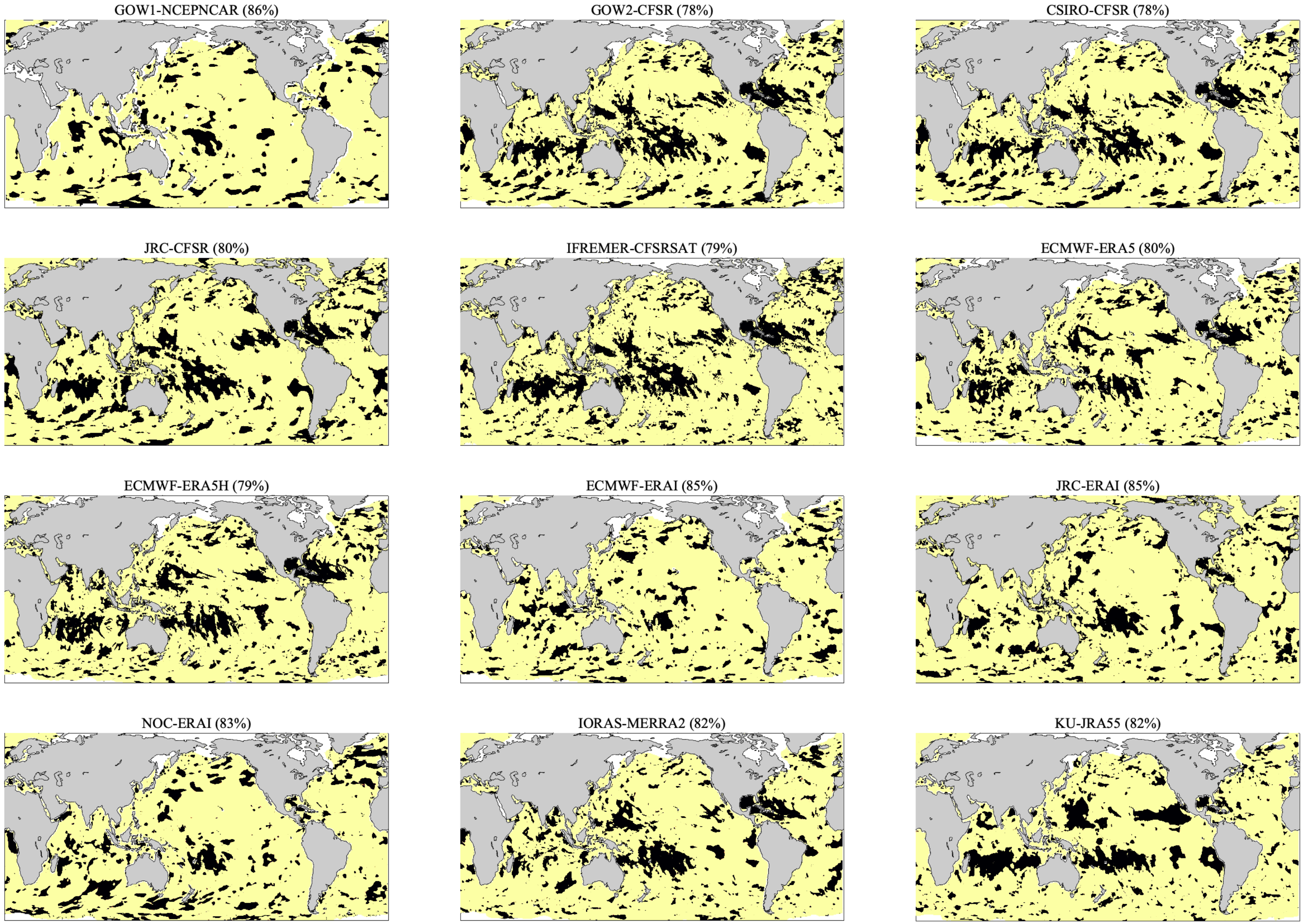

Supplementary Fig. S15 Results of BIC criterion (Methods). Yellow represents regions where BIC (GEV-AMAX) > BIC (GUM-AMAX). The percentage of global ocean area for which the BIC (GEV-AMAX) > BIC (GUM-AMAX) is shown on top of each panel.

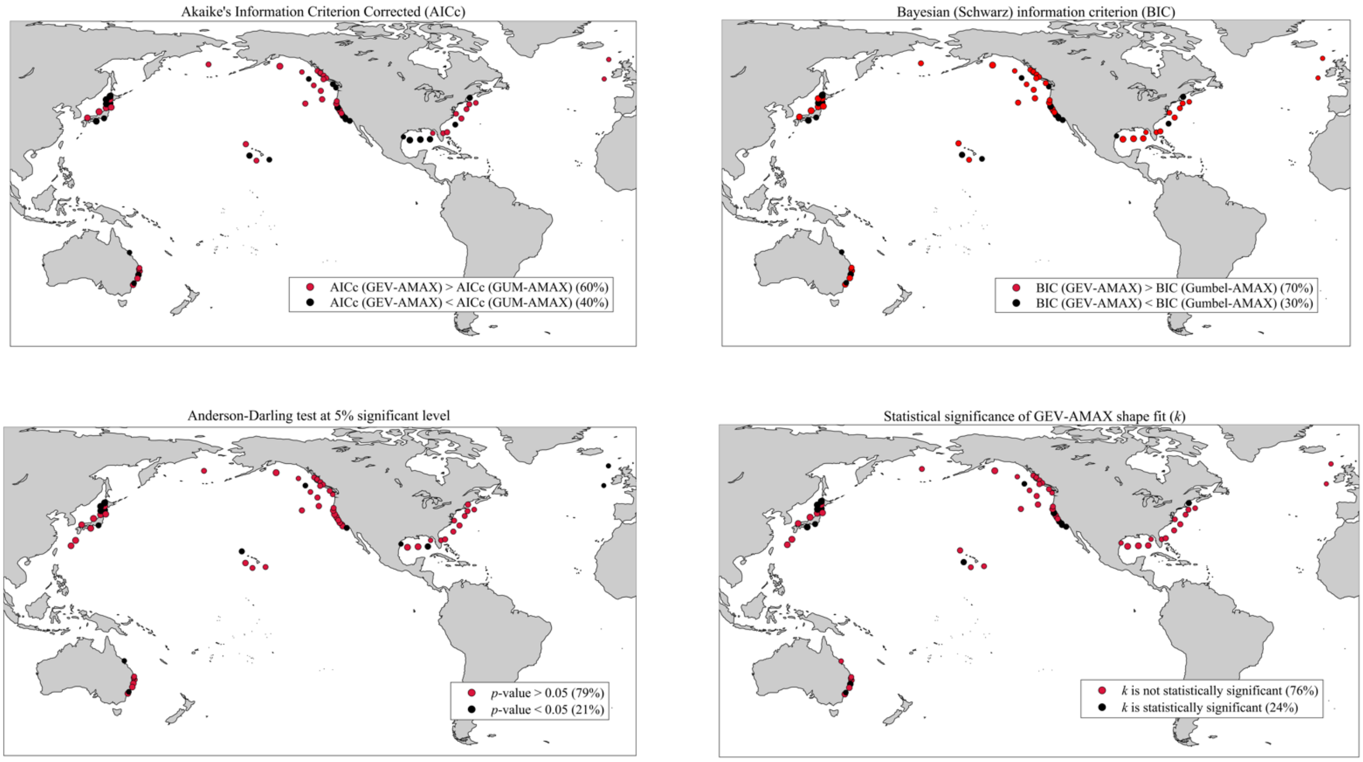

Supplementary Fig. S16 Aikake Information Criterion corrected (AICc), Bayesian information criterion (BIC), Anderson-Darling's (AD) test and significance of fit of shape parameter (*k*) for each wave buoy location, respectively.

Supplementary Table S1 – Summary of global wave model products used in this analysis.

Name of product (Temporal record)	Atmospheric Reanalysis[e] (Levels)	Assimilation System and Method	Spatial and Temporal Resolution	Sea-Ice Model	Wave Model	Source-terms ($\beta_{\max}$)	Spatial and Temporal Resolution	Spectral Frequency	Bathymetry Source	Bias Adjust.	Validation
ECMWF-ERA5 (1980-2015)	ERA5 (L137)	IFS 41R2/ 4D-VAR	0.25° / 1h	OSI-SAF (daily)	EC-WAM (CY41R2)	ST3 (default)	0.36° / 1h	30f x 24d	ETOPO2	Assimilation	Yes (buoys)
ECMWF-ERAI (1980-2015)	ERA-Interim (L91)	IFS 31R2/ 4D-VAR	0.75° / 6h	OSI-SAF (daily)	EC-WAM (CY31R2)	ST3 (default)	1.0° / 6h	30f x 24d	ETOPO2	Assimilation	Yes (buoys)
IHC-GOW1.0 (1980-2015)	NCEP/NCAR (L28)	GDAS/ 3D-VAR	1.90° x 1.87° / 6h	MOM3 (hourly)	WWIII v2.22	ST2 (default)	1.50° x 1.0° / 6h	25f x 72d	ETOPO2	Altimetry H_s	Yes (buoys)
ECMWF-ERA5H (1980-2015)	ERA5 (L137)	IFS 41R2/ 4D-VAR	0.25° / 1h	ERA5 (daily)	EC-WAM (CY46R1)	ST4 (default)	0.50° / 1h	36f x 36d	ETOPO1	None	Yes (buoys)
KU-JRA55 (1980-2012)	JRA55 (L60)	GSM- 4D-VAR	0.56° / 6h	COBE (monthly)	WWIII v4.18	ST4 (default)	0.56° / 1h	29f x 30d	ETOPO5	None	Yes (buoys)
IORAS-MERRA2 (1980-2015)	MERRA2 (L72)	GEOS-5- 3D-VAR	0.5° x 0.62° / 6h	MERRA2 (hourly)	WW3 V5.03	ST4 (default)	0.50 x 0.62° / 6h	32f x 24d	ETOPO2	None	Yes (buoys and VOS)
NOC-ERAI (1980-2015)	ERA-Interim (L91)	IFS 31R2/ 4D-VAR	0.75° / 6h	LIM2 (daily)	WWIII v3.14	ST2 (default)	0.70 x 0.47° / 1h	30f x 36d	ETOPO2	None	Yes (buoys)

JRC-ERAI (1980-2015)	ERA-Interim (L91)	4D-VAR (R2)	0.75° / 6h	None	WW3	ST4 (default)	1.0° / 12h	25f x 24d	ETOPO5	None	No
IFREMER-CFSRSAT (1980-2015)	CFSR (L64)	GDAS/ CFS-3D-VAR	0.50° / 1h	MOM4 (hourly)	WWIII v5.16	ST4 (β = 1.30)	0.50° / 1h	32f x 24d	ETOPO5	Altimetry U_{10}	Yes (buoys)
IHC-GOW2.0 (1980-2015)	CFSR (L64)	GDAS/ CFS-3D-VAR	0.50° / 1h	MOM4 (hourly)	WWIII v4.18	ST4 (β = 1.26)	0.25 / 0.50° / 1h	32f x 24d	ETOPO2	None	Yes (buoys)
CSIRO-CFSR (1980-2015)	CFSR (L64)	GDAS/ CFS-3D-VAR	0.50° / 1h	MOM4 (daily)	WWIII v4.08/4.18	ST4 (default)	1.0° / 0.40° / 1h	29f x 24d	DBDB2v3	None	Yes (buoys)
JRC-CFSR (1980-2015)	CFSR (L64)	GDAS/CFS-3DVAR	0.50° / 1h	None	WW3	ST4 (β = 1.52)	1.5° / 0.50° / 3h	25f x 24d	ETOPO5	None	No

Supplementary Table S2 Summary of global buoy network used in this analysis.

Region	Station ID (name)	Lon (°)	Lat (°)	Depth (m)	Length (years)	Final* (years)	Station ID (name)	Lon (°)	Lat (°)	Depth (m)	Length (years)	Final* (years)	Region
North America	NDBC-41001	287.37	34.60	4462	34	25	NDBC-46185	235.25	52.42	230	29	24	North America
	NDBC-41002	284.84	32.07	4297	33	24	NDBC-46204	237.56	51.37	222	32	26	
	NDBC-41009	279.80	28.50	45	27	22	NDBC-46206	232.06	48.83	72	29	27	
	NDBC-41010	281.45	28.90	887	27	25	NDBC-46207	227.57	50.86	2125	26	26	
	NDBC-42001	270.34	25.86	3217	35	32	NDBC-51001	197.86	23.94	4895	29	25	
	NDBC-42002	265.95	25.63	3125	35	32	NDBC-51002	202.24	17.11	4948	30	23	
	NDBC-42003	274.34	25.97	3292	35	29	NDBC-51003	199.34	19.18	1987	31	27	
	NDBC-42020	263.30	26.95	83	25	20	NDBC-51004	207.60	17.52	5183	30	23	
	NDBC-42036	275.48	28.50	52	21	20	IMOS-55481	153.63	-27.48	70	35	23	Australia
	NDBC-44004	289.50	38.47	190	29	23	IMOS-55017	153.71	-28.77	72	35	28	
	NDBC-44005	290.84	43.19	205	34	27	IMOS-55018	153.71	-30.34	72	35	30	
	NDBC-44008	290.66	49.50	63	32	24	IMOS-55019	152.85	-31.81	79	30	29	
	NDBC-44011	293.39	41.10	86	30	21	IMOS-55036	153.44	-27.96	69	28	23	
	NDBC-44014	285.17	36.58	47	25	22	IMOS-55022	151.02	-34.47	64	35	29	
	NDBC-46001	211.97	56.30	4214	35	33	IMOS-55024	151.41	-33.77	82	23	22	
	NDBC-46002	229.52	42.58	3438	33	27	IMOS-Mackay	149.54	-21.03	35*	35	20	
	NDBC-46004	224.00	51.00	3600	34	22	NOWPHAS-102	139.77	39.00	46	35	31	Japan
	NDBC-46005	228.98	46.07	2721	32	25	NOWPHAS-105	136.90	37.43	52	35	34	
	NDBC-46006	222.56	40.79	4294	33	25	NOWPHAS-201	139.91	40.65	51	35	34	
	NDBC-46011	239.10	34.90	464	34	27	NOWPHAS-202	141.42	40.92	45	35	34	
	NDBC-46012	237.12	37.36	206	35	26	NOWPHAS-305	132.03	34.09	50	35	30	

NDBC-46013	236.68	38.23	125	34	29	NOWPHAS-504	138.95	34.64	51	27	25
NDBC-46014	236.00	39.20	356	34	32	NOWPHAS-602	141.44	42.54	50	35	34
NDBC-46022	235.45	40.74	419	33	26	NOWPHAS-603	139.81	42.44	53	35	33
NDBC-46023	239.30	34.30	512	29	26	NOWPHAS-604	141.46	43.86	50	35	35
NDBC-46025	241.00	33.60	890	31	25	NOWPHAS-204	141.93	39.26	50	35	32
NDBC-46027	182.33	41.80	47	30	21	NOWPHAS-301	135.74	33.43	54	35	32
NDBC-46028	238.12	35.72	1075	32	27	MetOffice-K2	346.69	51.00	1994	24	18**
NDBC-46035	182.33	57.05	3682	29	24	MetOffice-K5	348.59	59.09	2011	21	18**
NDBC-46036	226.07	48.35	3548	28	22	Europe					
NDBC-46041	235.25	47.34	1645	28	20						
NDBC-46042	237.56	36.76	1890	28	26						
NDBC-46145	227.58	54.38	255	24	22						
NDBC-46146	236.27	49.34	45	23	22						
NDBC-46183	228.86	53.57	63	24	22						
NDBC-46184	226.06	53.90	3200	28	20						

*Number of years used for extreme value analysis after applying selection criteria (Methods).

**Number of years used for extreme value analysis after applying selection criteria is less than 20 years (European) (Methods).

Supplementary Table S2 – Summary of CMIP5-based global wave simulations used in this analysis.

Research Institution	Commonwealth Scientific and Industrial Research Organisation (CSIRO)	Joint Research Centre (JRC)*	National Oceanography Centre (NOC)	Environment and Climate Change Canada (ECCC)	IHE Institute for Water Education (IHE-DELFT)	Environment and Climate Change Canada (ECCC)
Country	Australia	UE	UK	Canada	Netherlands	Canada
Historical simulation	1979-2005	1979-2005	1970-2004	1979-2005	1979-2005	1950-2005
Future simulation	2080-2100	2010-2100	2005-2100	2081-2100	2006-2100	2006-2100
ACCESS1.0	■	■				■
ACCESS1.3		■				
BCC-CESM1.1	■			■		■
BCC-CESM1.1(m)						■
CanE77						■
CCSM4						■
CNRM-CM5	■					■
CSIRO-Mk3.6						■
EC-EARTH		■	■	■	■	■
FGOALS-s2						■
GFDL-CM3	■					
GFDL-E77M				■		■
HadGEM2-ES	■					
INMCM4	■			■		■
IPSL-CM5A-LR						■

MIROC-ESM						■
MIROC-ESM-CHEM						■
MIROC5	■			■		■
MPI-ESM-LR						■
MPI-ESM-MR						■
MRI-CGCM3	■					■
NorESM1-M						■
Number of forcing GCM (s)	8	3*	1	5	1	20
Wind-wave modelling method	Dynamical	Dynamical	Dynamical	Dynamical	Dynamical	Statistical
Statistical/Spectral wave model	WW3	WW3	WW3	WW3	WAM4.5	Regression
Surface/SLP forcing	3-hourly	3-hourly	3-hourly	3-hourly	3-hourly	6-hourly SLP
Atmospheric correction	-	-	-	-	-	SLP
Source-term package	ST3 (BJA)	ST4	ST4	ST4	ST3	-
Calibration	Default	Default	Default	Default	Default	-
Sea-Ice forcing	Monthly	No	Daily	Daily	Daily	-
Spatial resolution (°)	1 × 1	1.5 × 1.5	~0.7 × 0.5	1 × 1	1 × 1	1 × 1
Spectral partition	29f × 24d	25f × 24d	30f × 36d	29f × 24d	32f × 24d	-
Bathymetry data	ETOPO	ETOPO	GEBCO	DBDB2	ETOPO	-

*JRC ensemble provides 6 GCM wave simulations but we excluded three of them due to data issues leading to unrealistic estimates.

REFERENCES AND NOTES

1. P. L. Barnard, A. D. Short, M. D. Harley, K. D. Splinter, S. Vitousek, I. L. Turner, J. Allan, M. Banno, K. R. Bryan, A. Doria, J. E. Hansen, S. Kato, Y. Kuriyama, E. Randall-Goodwin, P. Ruggiero, I. J. Walker, D. K. Heathfield, Coastal vulnerability across the Pacific dominated by El Niño/Southern Oscillation. *Nat. Geosci.* **8**, 801–807 (2015).

2. N. Leonardi, N. Ganju, S. Fagherazzi, A linear relationship between wave power and erosion determines salt-marsh resilience to violent storms and hurricanes. *Proc. Natl. Acad. Sci. U.S.A.* **113**, 64–68 (2015).

3. A. Melet, B. Meyssignac, R. Almar, G. Le Cozannet, Under-estimated wave contribution to coastal sea-level rise. *Nat. Clim. Change* **8**, 234–239 (2018).

4. C. Storlazzi, S. B. Gingerich, A. van Dongeren, O. M. Cheriton, P. W. Swarzenski, E. Quataert, C. I. Voss, D. W. Field, H. Annamalai, G. A. Piniak, R. M. Call, Most atolls will be uninhabitable by the mid-21st century because of sea-level rise exacerbating wave-driven flooding. *Sci. Adv.* **4**, eaap9741 (2018).

5. K. A. Serafin, P. Ruggiero, H. F. Stockdon, The relative contribution of waves, tides, and nontidal residuals to extreme total water levels on U.S. West Coast sandy beaches. *Geophys. Res. Lett.* **44**, 1839–1847 (2017).

6. Z. Zhang, X.-M. Li, Global ship accidents and ocean swell-related sea states. *Nat. Hazards Earth Syst. Sci.* **17**, 2041–2051 (2017).

7. DNVGL, "Offshore standards DNVGL-OS-C201 - Structural design of offshore units" (2016).

8. ISO/TC 67/SC 7 Offshore structures, "ISO 19901-1:2015 Petroleum and natural gas industries — Specific requirements for offshore structures — Part 1: Metocean design and operating considerations" (2015).

9. A. B. Bugnot, M. Mayer-Pinto, L. Airoldi, E. C. Heery, E. L. Johnston, L. P. Critchley, E. M. A. Strain, R. L. Morris, L. H. L. Loke, M. J. Bishop, E. V. Sheehan, R. A. Coleman, K. A.

Dafforn, Current and projected global extent of marine built structures. *Nat. Sustain.* **4**, 33–41 (2021).

10. S. Gourvenec, F. Sturt, E. Reid, F. Trigos, Global assessment of historical, current and forecast ocean energy infrastructure: Implications for marine space planning, sustainable design and end-of-engineered-life management. *Renew. Sustain. Energy Rev.* **154**, 111794 (2022).

11. A. Toimil, I. J. Losada, R. J. Nicholls, R. A. Dalrymple, M. J. F. Stive, Addressing the challenges of climate change risks and adaptation in coastal areas: A review. *Coast. Eng.* **156**, 103611 (2020).

12. A. J. Dowdy, G. A. Mills, B. Timbal, Y. Wang, Fewer large waves projected for eastern Australia due to decreasing storminess. *Nat. Clim. Change* **4**, 283–286 (2014).

13. F. Ardhuin, J. E. Stopa, B. Chapron, F. Collard, R. Husson, R. E. Jensen, J. Johannessen, A. Mouche, M. Passaro, G. D. Quartly, V. Swail, I. Young, Observing sea states. *Front. Mar. Sci.* **6**, 124 (2019).

14. J. P. Sierra, M. Casas-Prat, Analysis of potential impacts on coastal areas due to changes in wave conditions. *Clim. Change* **124**, 861–876 (2014).

15. DNVGL, "Class Guideline DNVGL-CG-0130 - Wave loads" (2018).

16. R. Almar, R. Ranasinghe, E. W. J. Bergsma, H. Diaz, A. Melet, F. Papa, M. Vousdoukas, P. Athanasiou, O. Dada, L. P. Almeida, E. Kestenare, A global analysis of extreme coastal water levels with implications for potential coastal overtopping. *Nat. Commun.* **12**, 3775 (2021).

17. M. I. Vousdoukas, L. Mentaschi, E. Voukouvalas, M. Verlaan, S. Jevrejeva, L. P. Jackson, L. Feyen, Global probabilistic projections of extreme sea levels show intensification of coastal flood hazard. *Nat. Commun.* **9**, 2360 (2018).

18. S. Vitousek, P. L. Barnard, C. H. Fletcher, N. Frazer, L. H. Erikson, C. D. Storlazzi, Doubling of coastal flooding frequency within decades due to sea-level rise. *Sci. Rep.* **7**, 1399 (2017).

19. E. Kirezci, I. R. Young, R. Ranasinghe, S. Muis, R. J. Nicholls, D. Lincke, J. Hinkel, Projections of global-scale extreme sea levels and resulting episodic coastal flooding over the 21st century. *Sci. Rep.* **10**, 11629 (2020).

20. J. B. Shope, L. H. Erikson, P. L. Barnard, C. D. Storlazzi, K. Serafin, K. Doran, H. Stockdon, B. Reguero, F. Mendez, S. Castanedo, A. Cid, L. Cagigal, P. Ruggiero, Characterizing storm-induced coastal change hazards along the United States West Coast. *Sci. Data* **9**, 224 (2022).

21. S. Caires, A. Sterl, 100-Year return value estimates for ocean wind speed and significant wave height from the ERA-40 data. *J. Climate* **18**, 1032–1048 (2005).

22. J. Hinkel, L. Feyen, M. Hemer, G. Le Cozannet, D. Lincke, M. Marcos, L. Mentaschi, J. L. Merkens, H. de Moel, S. Muis, R. J. Nicholls, A. T. Vafeidis, R. S. W. van de Wal, M. I. Vousdoukas, T. Wahl, P. J. Ward, C. Wolff, Uncertainty and bias in global to regional scale assessments of current and future coastal flood risk. *Earth's Future* **9**, e2020EF001882 (2021).

23. J. E. Stopa, K. F. Cheung, Intercomparison of wind and wave data from the ECMWF reanalysis interim and the NCEP Climate Forecast System Reanalysis. *Ocean Model.* **75**, 65–83 (2014).

24. J. E. Stopa, F. Ardhuin, A. Babanin, S. Zieger, Comparison and validation of physical wave parameterizations in spectral wave models. *Ocean Model.* **103**, 2–17 (2016).

25. V. D. Sharmar, M. Y. Markina, S. K. Gulev, Global ocean wind-wave model hindcasts forced by different reanalyzes: A comparative assessment. *J. Geophys. Res. Oceans* **126**, e2020JC016710 (2021).

26. J. Morim, M. Hemer, X. L. Wang, N. Cartwright, C. Trenham, A. Semedo, I. Young, L. Bricheno, P. Camus, M. Casas-Prat, L. Erikson, L. Mentaschi, N. Mori, T. Shimura, B.

Timmermans, O. Aarnes, Ø. Breivik, A. Behrens, M. Dobrynin, M. Menendez, J. Staneva, M. Wehner, J. Wolf, B. Kamranzad, A. Webb, J. Stopa, F. Andutta, Robustness and uncertainties in global multivariate wind-wave climate projections. *Nat. Clim. Change* **9**, 711–718 (2019).

27. A. Meucci, I. R. Young, M. Hemer, K. Ebru, R. Roshanka, Projected 21st century changes in extreme wind-wave events. *Sci. Adv.* **6**, eaaz7295 (2020).

28. L. Mentaschi, M. I. Vousdoukas, E. Voukouvalas, A. Dosio, L. Feyen, Global changes of extreme coastal wave energy fluxes triggered by intensified teleconnection patterns. *Geophys. Res. Lett.* **44**, 2416–2426 (2017).

29. L. M. Bricheno, J. Wolf, Future wave conditions of Europe, in response to high-end climate change scenarios. *J. Geophys. Res. Oceans* **123**, 8762–8791 (2018).

30. M. Casas-Prat, X. L. Wang, Projections of extreme ocean waves in the arctic and potential implications for coastal inundation and erosion. *J. Geophys. Res. Oceans* **125**, e2019JC015745 (2020).

31. A. Patra, S.-K. Min, P. Kumar, X. L. Wang, Changes in extreme ocean wave heights under 1.5 °C, 2 °C, and 3 °C global warming. *Weather Clim. Extremes* **33**, 100358 (2021).

32. X. L. Wang, Y. Feng, V. R. Swail, Changes in global ocean wave heights as projected using multimodel CMIP5 simulations. *Geophys. Res. Lett.* **41**, 1026–1034 (2014).

33. H. Lobeto, M. Menendez, I. J. Losada, Future behavior of wind wave extremes due to climate change. *Sci. Rep.* **11**, 7869 (2021).

34. J. G. O'Grady, M. A. Hemer, K. L. McInnes, C. E. Trenham, A. G. Stephenson, Projected incremental changes to extreme wind-driven wave heights for the twenty-first century. *Sci. Rep.* **11**, 8826 (2021).

35. J. Morim, M. Hemer, N. Cartwright, D. Strauss, F. Andutta, On the concordance of 21st century wind-wave climate projections. *Glob. Planet. Change* **167**, 160–171 (2018).

36. M. Collins, M. Sutherland, L. Bouwer, S.-M. Cheong, T. Frölicher, H. Jacot Des Combes, M. Koll Roxy, I. Losada, K. McInnes, B. Ratter, E. Rivera-Arriaga, R. D. Susanto, D. Swingedouw, L. Tibig, Extremes, abrupt changes and managing risk, in *IPCC Special Report on the Ocean and Cryosphere in a Changing Climate* (Cambridge University Press, 2019), pp. 589–655. https://doi.org/10.1017/9781009157964.008.

37. J. Morim, L. H. Erikson, M. Hemer, I. Young, X. Wang, N. Mori, T. Shimura, J. Stopa, C. Trenham, L. Mentaschi, S. Gulev, V. D. Sharmar, L. Bricheno, J. Wolf, O. Aarnes, J. Perez, J. Bidlot, A. Semedo, B. Reguero, T. Wahl, A global ensemble of ocean wave climate statistics from contemporary wave reanalysis and hindcasts. *Sci. Data* **9**, 358 (2022).

38. E. Vanem, Uncertainties in extreme value modelling of wave data in a climate change perspective. *J. Ocean Eng. Mar. Energy.* **1**, 339–359 (2015).

39. S. Coles, *An Introduction to Statistical Modeling of Extreme Values* (Springer Series in Statistics book series (SSS), 2001).

40. S. Caires, “JCOMM Technical Report No. 57 - Extreme value analysis: Wave data” (2011).

41. R. M. Campos, J. H. G. M. Alves, C. Guedes Soares, L. G. Guimaraes, C. E. Parente, Extreme wind-wave modeling and analysis in the south Atlantic ocean. *Ocean Model.* **124**, 75–93 (2018).

42. A. Meucci, I. R. Young, Ø. Breivik, Wind and wave extremes from atmosphere and wave model ensembles. *J. Climate* **31**, 8819–8842 (2018).

43. K. Hodges, A. Cobb, P. L. Vidale, How well are tropical cyclones represented in reanalysis datasets? *J. Climate* **30**, 5243–5264 (2017).

44. M. Rohrer, S. Brönnimann, O. Martius, C. C. Raible, M. Wild, G. P. Compo, Representation of extratropical cyclones, blocking anticyclones, and alpine circulation types in multiple reanalyses and model simulations. *J. Climate* **31**, 3009–3031 (2018).

45. Z. S. Aarons, S. J. Camargo, J. D. O. Strong, H. Murakami, Tropical cyclone characteristics in the MERRA-2 reanalysis and AMIP simulations. *Earth Space Sci.* **8**, e2020EA001415 (2021).

46. G.-F. Bian, G.-Z. Nie, X. Qiu, How well is outer tropical cyclone size represented in the ERA5 reanalysis dataset? *Atmos. Res.* **249**, 105339 (2021).

47. K. I. Hodges, R. W. Lee, L. Bengtsson, A comparison of extratropical cyclones in recent reanalyses ERA-Interim, NASA MERRA, NCEP CFSR, and JRA-25. *J. Climate* **24**, 4888–4906 (2011).

48. T. Wahl, I. D. Haigh, R. J. Nicholls, A. Arns, S. Dangendorf, J. Hinkel, A. B. A. Slangen, Understanding extreme sea levels for broad-scale coastal impact and adaptation analysis. *Nat. Commun.* **8**, 16075 (2017).

49. J. Morim, C. Trenham, M. Hemer, X. L. Wang, N. Mori, M. Casas-Prat, A. Semedo, T. Shimura, B. Timmermans, P. Camus, L. Bricheno, L. Mentaschi, M. Dobrynin, Y. Feng, L. Erikson, A global ensemble of ocean wave climate projections from CMIP5-driven models. *Sci. Data* **7**, 105 (2020).

50. Z. Hausfather, G. P. Peters, Emissions – The ‘business as usual’ story is misleading. *Nature* **577**, 618–620 (2020).

51. R. J. Haarsma, M. J. Roberts, P. L. Vidale, C. A. Senior, A. Bellucci, Q. Bao, P. Chang, S. Corti, N. S. Fučkar, V. Guemas, J. von Hardenberg, W. Hazeleger, C. Kodama, T. Koenigk, L. R. Leung, J. Lu, J.-J. Luo, J. Mao, M. S. Mizielinski, R. Mizuta, P. Nobre, M. Satoh, E. Scoccimarro, T. Semmler, J. Small, J.-S. von Storch, High resolution model intercomparison project (HighResMIP v1.0) for CMIP6. *Geosci. Model Dev.* **9**, 4185–4208 (2016).

52. S. J. Camargo, Global and regional aspects of tropical cyclone activity in the CMIP5 models. *J. Climate* **26**, 9880–9902 (2013).

53. T. Shimura, N. Mori, H. Mase, Future projections of extreme ocean wave climates and the relation to tropical cyclones: Ensemble experiments of MRI-AGCM3.2H. *J. Climate* **28**, 9838–9856 (2015).

54. B. Timmermans, D. Stone, M. Wehner, H. Krishnan, Impact of tropical cyclones on modeled extreme wind-wave climate. *Geophys. Res. Lett.* **44**, 1393–1401 (2017).

55. C. M. Patricola, M. F. Wehner, Anthropogenic influences on major tropical cyclone events. *Nature* **563**, 339–346 (2018).

56. R. K. Hoeke, K. L. McInnes, J. C. Kruger, R. J. McNaught, J. R. Hunter, S. G. Smithers, Widespread inundation of Pacific islands triggered by distant-source wind-waves. *Glob. Planet. Change* **108**, 128–138 (2013).

57. M. Marcos, J. Rohmer, M. I. Vousdoukas, L. Mentaschi, G. le Cozannet, A. Amores, Increased extreme coastal water levels due to the combined action of storm surges and wind waves. *Geophys. Res. Lett.* **46**, 4356–4364 (2019).

58. M. I. Vousdoukas, J. Clarke, R. Ranasinghe, L. Reimann, N. Khalaf, T. M. Duong, B. Ouweneel, S. Sabour, C. E. Iles, C. H. Trisos, L. Feyen, L. Mentaschi, N. P. Simpson, African heritage sites threatened as sea-level rise accelerates. *Nat. Clim. Change* **12**, 256–262 (2022).

59. M. I. Vousdoukas, L. Mentaschi, E. Voukouvalas, M. Verlaan, L. Feyen, Extreme sea levels on the rise along Europe’s coasts. *Earth's Future* **5**, 304–323 (2017).

60. G. Dodet, A. Melet, F. Ardhuin, X. Bertin, D. Idier, R. Almar, The contribution of wind-generated waves to coastal sea-level changes. *Surv. Geophys.* **40**, 1563–1601 (2019).

61. I. A. Houghton, C. Hegermiller, C. Teicheira, P. B. Smit, Operational assimilation of spectral wave data from the Sofar Spotter network. *Geophys. Res. Lett.*, **49**, e2022GL098973. (2022).

62. J. E. Stopa, Wind forcing calibration and wave hindcast comparison using multiple reanalysis and merged satellite wind datasets. *Ocean Model.* **127**, 55–69 (2018).

63. W. Sasaki, Impact of satellite data assimilation in atmospheric reanalysis on the marine wind and wave climate. *J. Climate* **29**, 6351–6361 (2016).

64. P. Malakar, A. P. Kesarkar, J. Bhate, A. Deshamukhya, Appraisal of data assimilation techniques for dynamical downscaling of the structure and intensity of tropical cyclones. *Earth Space Sci.* **7**, e2019EA000945 (2020).

65. J.-E. Kim, S.-Y. Hong, A global atmospheric analysis dataset downscaled from the NCEP–DOE reanalysis. *J. Climate* **25**, 2527–2534 (2012).

66. S. Muis, M. Verlaan, H. C. Winsemius, J. C. J. H. Aerts, P. J. Ward, A global reanalysis of storm surges and extreme sea levels. *Nat. Commun.* **7**, 11969 (2016).

67. T. W. Anderson, D. A. Darling, A test of goodness of fit. *J. Am. Stat. Assoc.* **49**, 765–769 (1954).

68. H. Akaike, Canonical correlation analysis of time series and the use of an information criterion, in *Mathematics in Science and Engineering*, R. K. Mehra, D. G. Lainiotis, Eds. (Elsevier, 1976), vol. 126, pp. 27–96. www.sciencedirect.com/science/article/pii/S0076539208608693.

69. F. J. Méndez, M. Menéndez, A. Luceño, I. J. Losada, Analyzing monthly extreme sea levels with a time-dependent GEV model. *J. Atmos. Oceanic Tech.* **24**, 894–911 (2007).

70. J. G. O’Grady, A. G. Stephenson, K. L. McInnes, Gauging mixed climate extreme value distributions in tropical cyclone regions. *Sci. Rep.* **12**, 4626 (2022).

71. B. S. Everitt, *Cluster Analysis* (Wiley Series in Probability and Statistics, ed. 5, 2011).

72. J. Morim, S. Vitousek, M. Hemer, B. Reguero, L. Erikson, M. Casas-Prat, X. L. Wang, A. Semedo, N. Mori, T. Shimura, L. Mentaschi, B. Timmermans, Global-scale changes to extreme ocean wave events due to anthropogenic warming. *Environ. Res. Lett.* **16**, 074056 (2021).

73. T. Zhang, B. Tian, D. Sengupta, L. Zhang, Y. Si, Global offshore wind turbine dataset. *Sci. Data* **8**, 191 (2021).

74. C. Izaguirre, I. J. Losada, P. Camus, J. L. Vigh, V. Stenek, Climate change risk to global port operations. *Nat. Clim. Change* **11**, 14–20 (2021).

75. B. G. Reguero, M. Menéndez, F. Méndez, R. Mínguez, I. Losada, A global ocean wave (GOW) calibrated reanalysis from 1948 onwards. *Coast. Eng.* **65**, 38–55 (2012).

76. G. A. Smith, M. Hemer, D. Greenslade, C. Trenham, S. Zieger, T. Durrant, Global wave hindcast with Australian and Pacific Island focus: From past to present. *Geosci. Data J.* **8**, 24–33 (2021).

77. J. Perez, M. Menendez, I. Losada, GOW2: A global wave hindcast for coastal applications. *Coast. Eng.* **124**, 1–11 (2017).

78. J. E. Stopa, F. Ardhuin, E. Stutzmann, T. Lecocq, Sea state trends and variability: Consistency between models, altimeters, buoys, and seismic data (1979–2016). *J. Geophys. Res. Oceans* **124**, 3923–3940 (2019).

79. B. W. Timmermans, C. P. Gommenginger, G. Dodet, J.-R. Bidlot, Global wave height trends and variability from new multimission satellite altimeter products, reanalyses, and wave buoys. *Geophys. Res. Lett.* **47**, e2019GL086880 (2020).

80. J.-R., Bidlot, G. Lemos, A. Semedo, *2nd International Workshop on Waves, Storm Surges, and Coastal Hazards incorporating 16th International Workshop on Wave Hindcasting and Forecasting (2019)*, Melbourne, Australia, 10 to 15 November 2019.

81. T. Shimura, N. Mori, High-resolution wave climate hindcast around Japan and its spectral representation. *Coast. Eng.* **151**, 1–9 (2019).

82. D. P. Dee, S. M. Uppala, A. J. Simmons, P. Berrisford, P. Poli, S. Kobayashi, U. Andrae, M. A. Balmaseda, G. Balsamo, P. Bauer, P. Bechtold, A. C. M. Beljaars, L. van de Berg, J. Bidlot, N. Bormann, C. Delsol, R. Dragani, M. Fuentes, A. J. Geer, L. Haimberger, S. B.

Healy, H. Hersbach, E. V. Hólm, L. Isaksen, P. Kållberg, M. Köhler, M. Matricardi, A. P. Mc Nally, B. M. Monge-Sanz, J.-J. Morcrette, B.-K. Park, C. Peubey, P. de Rosnay, C. Tavolato, J.-N. Thépaut, F. Vitart, The ERA-Interim reanalysis: Configuration and performance of the data assimilation system. *Q. J. Roy. Meteorol. Soc.* **137**, 553–597 (2011).

83. H. Hersbach, B. Bell, P. Berrisford, S. Hirahara, A. Horányi, J. Muñoz-Sabater, J. Nicolas, C. Peubey, R. Radu, D. Schepers, A. Simmons, C. Soci, S. Abdalla, X. Abellan, G. Balsamo, P. Bechtold, G. Biavati, J. Bidlot, M. Bonavita, G. De Chiara, P. Dahlgren, D. Dee, M. Diamantakis, R. Dragani, J. Flemming, R. Forbes, M. Fuentes, A. Geer, L. Haimberger, S. Healy, R. J. Hogan, E. Hólm, M. Janisková, S. Keeley, P. Laloyaux, P. Lopez, C. Lupu, G. Radnoti, P. de Rosnay, I. Rozum, F. Vamborg, S. Villaume, J.-N. Thépaut, The ERA5 global reanalysis. *Q. J. Roy. Meteorol. Soc.* **146**, 1999–2049 (2020).
